# Genomic analysis of the secretion stress response in the enzyme-producing cell factory *Aspergillus niger*

**DOI:** 10.1186/1471-2164-8-158

**Published:** 2007-06-11

**Authors:** Thomas Guillemette, Noël NME van Peij, Theo Goosen, Karin Lanthaler, Geoffrey D Robson, Cees AMJJ van den Hondel, Hein Stam, David B Archer

**Affiliations:** 1 School of Biology, University of Nottingham, University Park, Nottingham, NG7 2RD, UK; 2 DSM Food Specialties, P.O. Box 1, 2600 MA Delft, The Netherlands; 3 Biocentre, HAN University, Laan van Scheut 2, 6525 EM Nijmegen, The Netherlands; 4 Faculty of Life Sciences, Stopford Building, University of Manchester, Oxford Road, Manchester M13 9PT, UK; 5 Clusius Laboratory, Leiden University, P.O. Box 9505, 2300 RA Leiden, The Netherlands; 6 Laboratoire de Microbiologie, UMR 77 Pathologie Végétale, Université d'Angers, 2 bd Lavoisier, 49045 Angers cedex, France

## Abstract

**Background:**

Filamentous fungi such as *Aspergillus niger *have a high capacity secretory system and are therefore widely exploited for the industrial production of native and heterologous proteins. However, in most cases the yields of non-fungal proteins are significantly lower than those obtained for fungal proteins. One well-studied bottleneck appears to be the result of mis-folding of heterologous proteins in the ER during early stages of secretion, with related stress responses in the host, including the unfolded protein response (UPR). This study aims at uncovering transcriptional and translational responses occurring in *A. niger *exposed to secretion stress.

**Results:**

A genome-wide transcriptional analysis of protein secretion-related stress responses was determined using Affymetrix DNA GeneChips and independent verification for selected genes. Endoplasmic reticulum (ER)-associated stress was induced either by chemical treatment of the wild-type cells with dithiothreitol (DTT) or tunicamycin, or by expressing a human protein, tissue plasminogen activator (t-PA). All of these treatments triggered the UPR, as shown by the expression levels of several well-known UPR target genes. The predicted proteins encoded by most of the up-regulated genes function as part of the secretory system including chaperones, foldases, glycosylation enzymes, vesicle transport proteins, and ER-associated degradation proteins. Several genes were down-regulated under stress conditions and these included several genes that encode secreted enzymes. Moreover, translational regulation under ER stress was investigated by polysomal fractionation. This analysis confirmed the post-transcriptional control of *hacA *expression and highlighted that differential translation also occurs during ER stress, in particular for some genes encoding secreted proteins or proteins involved in ribosomal biogenesis and assembly.

**Conclusion:**

This is first genome-wide analysis of both transcriptional and translational events following protein secretion stress. Insight has been gained into the molecular basis of protein secretion and secretion-related stress in an effective protein-secreting fungus, and provides an opportunity to identify target genes for manipulation in strain improvement strategies.

## Background

Many species of filamentous fungi such as *Aspergillus niger *are effective secretors of, mainly, hydrolytic enzymes to facilitate their saprophytic lifestyles by providing substrates from polymeric organic materials. This high capacity secretory system has driven the exploitation of filamentous fungi as cell factories for provision of enzymes used in a wide variety of applications [1]. Since the availability of gene-transfer systems, several fungal species have become potentially excellent hosts for achieving commercial yields of heterologous proteins. However, the yields of recombinants enzymes are often lower than desired, and this is especially so when the donor organism is not a fungus [2]. Many approaches have been used to overcome the bottlenecks to achieving high-secreted yields of heterologous proteins from fungi [2,3] but the levels do not reach the same level as the best native protein.

Several data suggest that bottlenecks mainly exist at the post-transcriptional level, and most probably within the secretory pathway [4,5]. The ER orchestrates the folding and some post-translational modifications of proteins that reside in, or pass through, the endomembrane system of a eukaryotic cell. In expression systems, the large flux of proteins being translocated into the ER generates a need to enhance the efficiency of protein folding and transport as well as the quality control of the synthesized proteins. Increased flux of proteins through the ER, especially those which do not fold correctly, or at least with the required kinetics, lead to the induction of stress responses that are collectively called protein secretion stress or ER stress. Mechanisms that allow the cell to sense the state of the lumen and to respond to ER stress conditions have been characterized in detail in the yeast *Saccharomyces cerevisiae *and in mammalian cells [6]. The first of these mechanisms is the unfolded protein response (UPR), a complex intracellular signaling pathway that increases the transcriptional activity of a number of genes involved in protein folding, glycosylation and transport [6,7]. The UPR also appears to be intimately linked to the ER-associated (protein) degradation (ERAD) pathway [7,8], in which misfolded proteins in the ER lumen are retrotranslocated (dislocated) through the translocon to the cytoplasm, and are ubiquitinated and degraded by the proteasome [9]. Recently, a novel type of feed-back mechanism, termed repression under secretion stress (RESS), has been suggested in filamentous fungi [10,11]. It functions during secretion stress and down-regulates the transcript levels of some genes. A gene array study under ER stress conditions has recently been published with *A. nidulans *where ca. 30% of the predicted genes were represented on the array [12]. They described the first attempt to analyze in part the influence of the production and secretion of a heterologous protein on the cellular transcript profile of a filamentous fungus. A complete analysis of ER stress responses in the yeast *S. cerevisiae*, a fungus that does not secrete proteins as effectively as *A. niger *has also been described [7].

The ER surveillance system continuously coordinates the activity and participation of the processing and degradation pathways for unfolded proteins. Upon accumulation of unfolded proteins in the ER lumen, the UPR is activated, reducing the amount of new protein translocated into the ER lumen, increasing dislocation of proteins from the lumen of the ER and, then, their degradation, and bolstering the protein-folding capacity of the ER. Thus, understanding the process of folding and the stress responses in filamentous fungi may hold the major key to improving their use as cell factories for production of recombinant proteins. A genome-wide expression analysis of these secretion-related stress responses has never been reported in industrially-exploited species, since genome sequence data has not previously been available. Recently, the sequencing and annotation of the genome of *A. niger *have been completed [13]. DNA GeneChips have been made available and provide an unprecedented resource for exploring expression profiles in response to particular environmental cues. Here, we report the first gene expression analysis studies showing the response to various secretion stresses and assessing the breadth of the UPR in *A. niger*.

## Results

### Transcriptional analysis of the ER stress response

To gain further insight into the UPR of filamentous fungi, we identified transcriptional targets of this signaling pathway in *A. niger *by monitoring mRNA levels using oligonucleotide arrays on Affymetrix GeneChips. The UPR was induced by treating mycelium cultures for 2 h with two chemical agents which disrupt protein folding in the ER. Dithiothreitol (DTT) is a strong reducing agent that prevents disulfide bond formation. Tunicamycin is a drug that inhibits N-linked glycosylation by preventing core oligosaccharide addition to nascent polypeptides and thereby blocks protein folding and transit through the ER [14]. Finally, a strain [15] producing recombinant tissue plasminogen activator (t-PA), a serine protease, was chosen to assess the UPR under conditions of heterologous protein production. Previous studies indicated that the expression of t-PA in *A. niger *leads to the appearance of the spliced form of *hacA *mRNA (*hacA*^*i*^) (which is translated to yield the UPR-mediating transcription factor HacA) and the simultaneous up-regulation of *bipA *and *pdiA *[15,16]. Unlike with *S. cerevisiae *[7] it was not possible to include strains of *A. niger *that are devoid of genes encoding key mediators of the UPR such as *ireA *and *hacA *because such strains are not available despite the cloning and functional characterization of those genes [7,17]. Deletion of *ireA *in *A. niger *has not been achieved and the only Δ*hacA *strain described to date grows very differently from the wild-type strain of *A. niger *(H. Mulder, personal communication) and, anyway, is not generally available.

**Figure 1:**
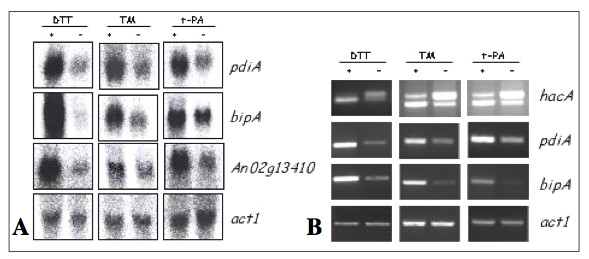
GeneChip results were confirmed for some genes using Northern blotting and RT-PCR. Examples are provided for both Northern blots (A) and RT-PCR (B). Note that the RT-PCR for the *hacA *mRNA was designed to indicate enhanced splicing of the *hacA *mRNA intron under stress conditions (DTT, tunicamycin and production of t-PA). This is shown as a relative increase in the amount of the processed (lower band) *hacA*^*i *^form of the mRNA compared to the unprocessed higher band (*hacA*^*u*^). Probing or PCR for an actin gene was used as a non-stress-responsive control transcript.

Replicate experiments were performed for each of the three stress responses. All (experimental and control) chips were normalized/scaled to a target intensity of 100 by global scaling (details regarding Scaling and Normalization are listed in the Affymetrix Microarray Suite User Guide Version 5.0, Appendix D) with scaling factors being comparable for all comparisons [see Additional file 1]. Next, *intra*-experiment and *inter*-experiment GeneChip comparisons were processed for each of the 3 stress conditions and all genes with a detected transcript in at least one condition were checked for even distribution of intensities. For all the probe sets on the Affymetrix GeneChip, we determined the fold change in expression due to each treatment by comparing its expression level in the treated sample to its level in the untreated control. Two comparisons were processed for each of the 3 stress conditions. Increased expression of *bipA, pdiA *(two genes expected to be up-regulated by ER stress) and an uncharacterised *A. niger *gene (An02g13410, putative homolog of an acetyl CoA transporter) shown on the GeneChips to be up-regulated, was confirmed in response to both chemical treatments and to t-PA production by Northern hybridisation (Fig. 1A). Increased levels of *hacA*^*i *^mRNA was also confirmed by RT-PCR across the *hacA *mRNA intron, and RT-PCR was also used to confirm both the GeneChip and Northern hybridisation data showing that the mRNA levels of *bipA *and *pdiA *were increased under stress (Fig. 1B). For tunicamycin treatment especially but also t-PA expression, overall variations in gene expression levels were generally small, whereas DTT treatment showed a relatively large reponse [see Additional files 2, 3, 4]. With standard criteria of a fold change of 2, less than 10 differentially-expressed genes would be detected in the tunicamycin treatment and therefore selection criteria were adjusted to a less stringent 1.5-fold change for all treatments. According to the analytical criteria adopted (at least 1.5-fold change), a list of induced genes was produced with 79 independent entries for the heterologous protein production condition, 38 entries for the tunicamycin treatment and 865 entries for the DTT treatment. The repressed gene sets identified with these restrictions constituted 110, 11 and 774 independent entries from the t-PA producing strain and for the tunicamycin and DTT treatments respectively. Tables 1 and 2 present the lists of genes that were up-regulated or down-regulated by at least 2 of the 3 treatments respectively. A more complete list of genes differentially expressed following each treatment (tunicamycin and t-PA production) is provided in the supplementary information [see Additional files 5 and 6]. Moreover, an additional GeneChip experiment was conducted after a 1 h exposure to tunicamycin and results are also included in those Tables.

### Comparisons of ER stress induced under different conditions

**Figure 2:**
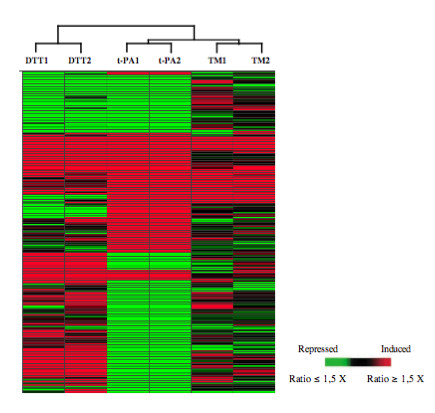
Hierarchical clustering of records in a dendrogram (tree graph) based on the similarity of the signal log ratios obtained in each of the duplicate stress studies. Records (188) were selected based on differential expression in the tPA comparisons. This tree is representative for multiple clusterings performed using signals or signal log ratios. The genes have been rearranged into their cluster order and are represented on the vertical axis. The experiments are represented on the horizontal axis. The significance of the colour scale is indicated.

**Figure 3:**
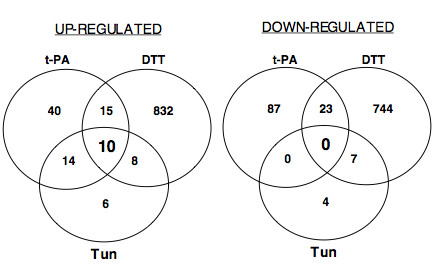
Venn diagrams of the numbers of overlapping and non-overlapping induced (≥ 1.5 fold) or repressed (≤ 1.5 fold) genes on the *A. niger *array after exposure to DTT or tunicamycin (Tun) and in the t-PA producing strain (t-PA).

Comparison of the signal intensities on the GeneChips showed that, while the replicates for each stress condition clustered closely together, the DTT stress was more distant from the t-PA and tunicamycin stresses which were more closely clustered together (Fig. 2). The numbers of genes that were induced or repressed under each of the conditions, together with a summary of those regulated in more than one condition, are shown in Fig. 3. Of the up-regulated genes, only ten were found in all three conditions and these are indicated in Table 1. This list is dominated by genes that are expected to be directly influenced by the UPR, e.g. *pdiA, prpA, bipA, clxA*, and *lhsA*. It is striking that the majority of the DTT-induced genes were not anticipated ER stress genes and we conclude that, although DTT can induce the expression of a large number of genes it is not the most appropriate stress agent for studies of ER stress, a conclusion reached independently elsewhere [12,18]. In contrast, most of the tunicamycin-induced genes are also induced by t-PA and/or DTT and about half of the t-PA-induced genes are also induced by either DTT or tunicamycin. As previously reported in yeast [7], our results show that ER stress responses affect multiple ER and secretory pathway functions. As expected, we observe induction of ER-resident chaperones and other proteins involved in protein folding. However, these represent only a fraction of this set of target genes and we also found several categories of induced genes with other functions throughout the secretory pathway including translocation, protein glycosylation, vesicular transport, ER-associated degradation and lipid metabolism.

Fewer genes were down-regulated overall than were up-regulated and there were no genes down-regulated in all conditions. Exposure to tunicamycin for 2 h caused down-regulation of only eleven genes in total compared to 38 up-regulated. Over one hundred genes (t-PA) and nearly 800 genes (DTT treatment) were down-regulated (Table 2) and 23 of these were common to both the t-PA and DTT conditions. Among the 61 genes that we found to be down-regulated by more than one stress condition, we identified 30 sequences containing a signal peptide, 6 genes encoding extracellular proteins and 4 genes related to the cell wall. At least 5 other genes involved in cell wall biosynthesis or function were additionally repressed by t-PA production or tunicamycin treatment.

### Translational regulations during the ER stress response

ER stress is known to lead to differential up- and down-regulation of transcription, but differential translation has yet to be explored in filamentous fungi although a few reports are available with *S. cerevisiae *[19,20]. A direct analysis of translational control can be achieved by fractionation of cytoplasmic extracts in sucrose gradients, based on the methods described for polysome analysis [21], which involves size separation of large cellular components and monitoring the A_254 _across the gradient. This method enables the separation of free mRNPs (ribonucleoprotein particles) from mRNAs fully loaded with ribosomes (i.e. polysomes). As only polysomes represent actively translated transcripts, this fraction should be directly correlated with the set of *de novo *synthesized proteins in a particular cellular state and enables the determination of the translation efficiencies, which are characteristic for each transcript in a cell [22]. In addition, changes in the distribution of a given mRNA indicate how this translational efficiency can vary under different conditions. Because it is generally accepted that translational control predominantly occurs at the initiation step [23], the number of mRNA molecules engaged in polysomes should be a robust indicator of the synthesis rate of the corresponding protein.

**Figure 4:**
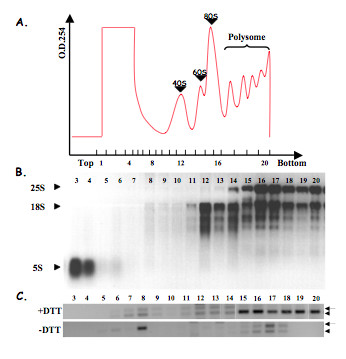
(A) Representative absorbance profile for RNA separated by velocity sedimentation through a 15–60% sucrose gradient. Fractions are numbered from the top to the bottom of the gradient. (B) RNA was extracted from each fraction and subjected to electrophoresis through a formaldehyde gel. The ribosomal RNA distribution profile (25S, 18S and 5S rRNA; indicated by arrowheads) enables the the assignment of OD_254 _peaks, corresponding to the 40S and 60S ribosomal subunits and to intact ribosomes (80S). (C) RT-PCR analysis with *hacA *primers were performed from each fraction of collected gradients from treated and non-treated cells. The full length *hacA*^*u *^mRNA (arrow) as well as low-molecular-weight version of *hacA*^*i *^(arrowhead) can be visualized.

Cytoplasmic extracts from DTT treated or untreated cells were loaded onto sucrose gradients and twenty fractions were collected from each one. RNA was extracted from each fraction and an aliquot was subjected to electrophoresis through a formaldehyde gel (Fig. 4B). As expected, 25S and 18S ribosomal RNAs were the prominent species. As described [21,24], the assignment of OD_254 _peaks corresponding to the 40S and 60S subunits and to intact ribosomes was confirmed with 18S and 25S RNAs distribution (Fig. 4A). There was no marked net change in the absorbance profile of DTT-treated samples compared to the control, indicating that the ER stress did not cause a global change of translational activity.

Furthermore, the distribution of spliced (*hacA*^*i*^) and unspliced *hacA *mRNA (*hacA*^*u*^) over the gradients was analysed by RT-PCR with *hacA *primers amplifying a fragment across the 20 nt intron region (Figure 4C). In *A. niger *control samples, a low level of *hacA*^*u *^transcripts was detected, which sedimented with both polysomal and non-polysomal fractions. Low level amplifications from the spliced form were also obtained in the polysomal fractions, suggesting that splicing occurs to a low level even in non-stressed samples. In DTT-treated samples, spliced and unspliced transcripts were detected but the spliced form was clearly predominant and was mainly recovered from the bottom of the gradient and therefore was ribosome-associated.

RNA fractions were pooled into non-polysomal and polysomal samples according to OD_254 _profiles and were used as a matrix for GeneChip hybridisation. Translational regulation of each mRNA was assessed by measuring the relative proportions of each mRNA in the polysomal and non-polysomal RNA fractions and then determining changes to this ratio. The ratio was determined by calculation of "DTT-induced shift from non-polysomal to polysomal samples": for each transcript microarray expression values were analysed by calculating (DTT-treated polysomal/DTT-treated non-polysomal)/(control polysomal/control non-polysomal). Several of these translational regulated genes are listed in the Table 3. Twenty six genes showed enhanced translation during DTT-treatment (a > 2 fold shift from non-polysomal to polysomal fractions). Several of the predicted proteins function as part of the secretory system including a signal peptidase and a proteasome protein. Two hundred and fifty three genes showed reduced translation (a < 2 fold shift from polysomal to non-polysomal fractions) including 108 hypothetical protein encoding genes. DTT treatment resulted in translational repression of a large number of genes with functions in ribosomal biogenesis and assembly. Included were several mRNAs encoding both large and small ribosomal subunit proteins (RPL and RPS families). This result may support a hypothesis of a translational repression as a mechanism of reducing ER throughput during ER stress. Three RPS and 2 RPL encoding genes are included in Table 3, but 9 other RPS and 23 other RPL were affected by this regulation, suggesting that RP mRNAs were coordinately regulated at the translational level. We also found that several mRNAs encoding secreted proteins were also redistributed from polysomes to monosomes and untranslated mRNPs. These results suggest that, in addition to the transcriptional repression mechanism called RESS, another feed-back mechanism could occur upon ER stress at the translational level and lead to a reduced amount of new protein translocated into the ER lumen. Our results also indicate that DTT repressed the translational activity of a number of genes belonging to other functional categories including the ERAD pathway, lipid metabolism or cell wall biogenesis (Table 3).

## Discussion

This is the first complete analysis of ER stress in *A. niger*. It has been made possible by the availability of the complete annotated genome sequence [13] and genome-wide GeneChips, which include built-in control sequences. The global ER stress response has already been reported in *S. cerevisiae *[7] but equivalent studies in the filamentous fungi have been hampered by the lack of complete genome sequences and the non-availability of gene arrays. Such bottlenecks are being removed and we have already seen the description of ER stress responses in *Trichoderma reesei *[25] and *A. nidulans *using arrays that cover approximately one third of the predicted open reading frames [12]. *A. nidulans *is scarcely exploited for its capacity to secrete enzymes because other filamentous fungi, including *A. niger*, have proved to hold advantages in terms of the range and yield of secreted enzymes. Previous analyses of secretion stress in *A. niger *have relied either on analysis of specified target genes [2,26] or have used cDNA subtraction libraries [18,27] which, in contrast to GeneChips, reveal only a small fraction of differentially expressed genes.

A low variation (a technical standard deviation of 0.16 and a biological standard deviation of 0.25 on average for the signal log ratio of 'present' genes) was shown for GeneChip replicates (data not shown). Generally, dependent on growth conditions, 5000–6000 genes were found to be expressed, with 10–20 genes detected as false positives in a single experiment. In any global transcriptomic study it is essential to have biological replicates that provide assurance of the validity of the conclusions reached. The biological replicates used in this study showed a small variation compared to the experimentally induced variation except for the tunicamycin study where the experimentally induced variation was small. Both for the chemostat-cultivated tPA strain and the shake-flask-cultivated tunicamycin-exposed cells, less than 4% and 1% of the expressed genes, respectively, were determined as changed by at least 1.5-fold.

We confirmed that each of the stress conditions led to induction of the UPR as judged by the transcriptional induction of *A. niger *genes known to be affected by ER stress, *bipA *[28] and *pdiA *[29]. In addition, we confirmed splicing of the 20bp intron in the *A. niger hacA *mRNA [10,16]. It has recently been established that the UPR does not just involve the simple switch based around the synthesis of the mediating transcription factor but that there is further complexity under some conditions [30-32]. In *A. niger *[10], *Trichoderma reesei *[11] and *S. cerevisiae *[33] it is known that ER stress can lead to the transcriptional down-regulation of some genes encoding secreted proteins and this effect, termed repression under ER stress (RESS) [11], may be independent of the UPR [10]. ER stress due to over-expression of membrane proteins can elicit the transcriptional up-regulation of *bipA *without apparent splicing of the *hacA *intron in *A. niger *[34]. This effect, together with RESS, indicates that there is complexity in ER stress responses in filamentous fungi. Our results confirm the existence of the RESS mechanism in *A. niger *since the transcription of several major secreted proteins encoding genes was clearly repressed by at least 2 treatments. Moreover, in common with the data generated in *Arabidopsis thaliana *[35], many repressed genes in *A. niger *encode membrane proteins and transporter proteins that may be essential for the maintenance of cellular ion homeostasis. Thus, the list presented in the additional file 6 contains several putative zinc, iron, calcium and manganese transporters and the zinc-regulated transcription factor Zap1, which were mainly repressed by t-PA production. Previous studies showed that both calcium [36] and zinc [37] are required for ER function in yeast and that their deficiency induces the UPR.

**Figure 5:**
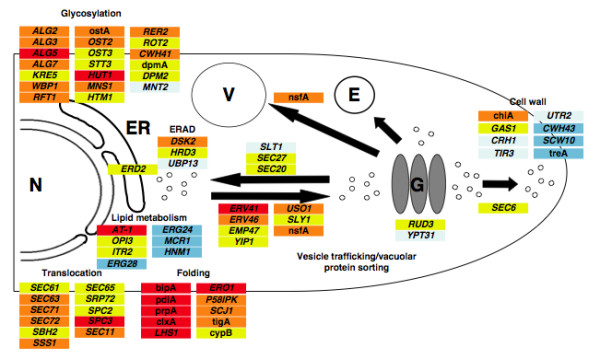
Model of the secretory pathway under different ER stress conditions (t-PA secretion, tunicamycin and DTT) together with examples of genes that are transcriptionally induced or repressed. The *A. niger *gene designation is provided where previously known or, otherwise, the *S. cerevisiae *gene name is provided. Red, genes up-regulated by 3 conditions; orange, genes up-regulated by 2 conditions; yellow, genes up-regulated by 1 condition; light blue, genes down-regulated by 1 condition; blue, genes down-regulated by 2 conditions. N, nucleus; ER, endoplasmic reticulum; E, endosome; V, vacuole; G, Golgi. ERAD is ER-associated degradation.

Analysis of the impact of secretion stress on the genes encoding components of the secretory system in *A. niger *is summarized in Fig. 5. Few of the translocon genes (encoding components of both the co- and post-translational translocation system), which were largely not represented in the *A. nidulans *arrays [12], were induced under two or more stress conditions. The signal sequence recognition system was transcriptionally unaffected except in one component but there was more response from components of the signal peptidase complex. Several of the translocon genes and one component of signal peptidase were induced under UPR in *S. cerevisiae *[7]. *A. niger *homologues of the *S. cerevisiae SEC11, SPC2 *and *SPC3 *were all up-regulated under ER stress. Of the predicted ER-resident chaperones, *bipA *was induced under all conditions as expected [28]. The calnexin-encoding *clxA *gene [38], where the production of prochymosin was previously shown to induce its transcription, was shown here to be additionally induced by both DTT and tunicamycin. A homolog of the *S. cerevisiae LHS1 *gene, *lhsA*, has not been previously described in *A. niger *but was annotated in the genome of *A. niger *and represented on the GeneChips. As with *bipA*, *lhsA *was transcriptionally up-regulated under all 3 conditions compared to controls. In *S. cerevisiae*, the chaperone cycle involves products encoded by *KAR2 *(encodes a Bip-like chaperone) and *LHS1 *(where the ATPase activities of these two Hsp70p chaperones are coordinately regulated) as well as nucleotide exchange activity provided by Sil1p and DNAJ proteins such as Scj1p and Jem1p [39]. The ER-resident chaperone and foldase system was generally induced under UPR conditions in *S. cerevisiae *even if *KAR2 *and *PDI1 *failed to meet the stringent criteria applied [7]. Although *lhsA *has been identified here as a stress-responsive gene, as was at least one DNAJ protein, no candidate genes have been found for the nucleotide exchange factor homolog of yeast *SIL1*. On the presumption that this functionality exists, it may reside in a protein with low sequence identity to Sil1p or in another component, e.g. LhsA. A putative ortholog of a mammalian p58-encoding gene (similarity to human p58 with e value of 4e-59, showing 32% amino acid identity over 450 residues) was induced by both t-PA and tunicamycin. P58 is involved in translational regulation in mammalian cells, its induction is mediated by ATF6 and it plays a role in regulating the PERK/eIF2alpha/ATF4 pathway [40]. Homologs of PERK and ATF4/6 appear to be absent from *A. niger *so the role of the putative p58 in *A. niger *is intriguing.

Manipulations of the ER lumenal environment have been previously examined in *Aspergillus *with the aim of improving the secreted yield of heterologous proteins. This has been attempted with individual genes such as *bipA *[41] and *pdiA *[42] as well as through manipulation of the UPR [43]. Detailed knowledge of the responses of individual genes to different stresses should permit refinements to these approaches that would be more consistently beneficial. The complexity of the chaperone cycle has already been mentioned but the formation of disulfide bonds is another key example. Although the *pdiA *gene encodes the principal foldase in *A. niger *[29], other genes encoding members of this foldase/isomerase family have also been described [44,45]. A fuller description of their responses to different stresses is provided by the GeneChip studies described here and shows that all three foldase genes are up-regulated by all stresses except for *tigA *by DTT. The contributions to foldase activity provided by the three identified lumenal foldases (PdiA, TigA, PrpA) is not known although, in *S. cerevisiae*, Pdi1p is known to contribute more activity than the other yeast foldases [46]. In *S. cerevisiae*, Ero1p is an essential lumenal protein involved in electron transfer during the formation of disulfide bonds and its homolog in *A. niger, eroA*, was induced under all stress conditions. Interestingly, Ero1p is retained in the lumen by attachment to the ER membrane in a manner that is not wholly understood [47] whereas the *A. niger *EroA is predicted to contain a conventional C-terminal ER retention sequence. The receptor for retention of C-terminal (H/K)DEL-containing proteins (Erd2p in *S. cerevisiae*) was induced under ER stress in both *A. niger *and *S. cerevisiae *[7]. In other areas, several genes involved in glycosylation of secretory proteins were induced under ER stress in both *S. cerevisiae *[7] and in *A. niger *(Fig. 5) and included a homolog of the yeast *RFT1 *gene that is responsible for translocation of lipid-linked glycan intermediates into the ER [48].

In fungi, the only reported translational control during the UPR concerns the expression of the yeast transcription factor Hac1p [20]. This control is mediated by a base-pairing interaction between an intron at the 3'end and the 5' untranslated region, which represses mRNA translation. Splicing of this unconventional intron is sufficient to relieve this translational block. It has been reported that the *HAC1*^*u *^mRNA in yeast was stable, located in the cytosol and associated with ribosomes, yet did not produce protein, indicating that the ribosomes engaged on the mRNA were stalled and that translation was attenuated at the elongation step [20]. Our results showed that *hacA*^*u *^mRNA could also sediment in non-polysomal fractions, suggesting that translation of this mRNA species may be blocked at the translational initiation step in addition to the previously reported elongation step, as also shown in yeast [49]. DTT treatment resulted in translational repression of a large number of genes with functions in ribosomal biogenesis and assembly (Table 3). This result may support the hypothesis of a translational repression as a mechanism of reducing ER throughput. It is known that the abundance of RP mRNAs rapidly decreases when yeast cells encounter stress situations [50,51]. We also found that several mRNAs encoding secreted proteins were also redistributed from polysomes to monosomes after DTT exposure and the gene *glaA *encoding glucoamylase, which is a major secreted protein in *A. niger*, was one of them. Therefore, in addition to the transcriptional down-regulation of *glaA *due to DTT stress, our results suggest that a post-transcriptional regulatory mechanism negatively affects the translation of *glaA *mRNA in *A. niger*. For the first time in filamentous fungi, these results suggest the existence, in addition to the RESS, of another feed-back mechanism that occurs upon ER stress at the translational level and leads to a reduction in the amount of new protein translocated into the ER lumen.

## Conclusion

This is the first complete analysis of ER stress in *A. niger*, a filamentous fungus used commercially for the secreted production of a range of native and heterologous proteins. It has been made possible by the availability of the complete annotated genome sequence and genome-wide GeneChips. We induced ER stress either by chemical treatments of the wild-type cells or by expressing a heterologous protein. Following the induction of ER stress, *A. niger *cells display a diverse array of adaptative changes in gene expression at both the transcriptional and translational levels. The transcriptional responses to each stress were compared and the overlaps common to these conditions led to the identification of robust sets of induced or repressed genes Thus, a range of transcriptional targets has been identified as putative candidates for secretion stress- related genes, including a large number of genes encoding components of the secretory system. Moreover, by combining polysomal fractionation with DNA GeneChip analyses, we have focused on the translational activity of several individuals mRNAs following DTT exposure. Although the observed polysome profiles were similar under control and ER stress conditions, we confirmed the post-transcriptional control of *hacA *expression and highlighted that differential translation could also occur during ER stress at least for some genes encoding secreted or ribosomal proteins. Many of the genes identified in this study provide targets for improving *A. niger *as a cell factory for protein production.

## Methods

### Strains, culture conditions and treatments

The AB4.1 strain, a *pyrG*^- ^strain that is auxotrophic for uridine [52], was used for the chemical treatment experiments. A protease-deficient strain (D15) of *A. niger*, derived from strain AB4.1, was used as a host for the production of human tissue plasminogen activator (t-PA). The t-PA was produced in the recombinant strain D15#25 as a fusion protein with the catalytic domain of the native glucoamylase protein. The gene encoding this fusion protein was expressed under control of the constitutive promoter for glyceraldehyde-3-phosphate dehydrogenase (*gpdA*).

The *A. niger *strains were maintained on potato dextrose agar slopes (Oxoid). All AB4.1 cultures were supplemented by 10 mM uridine (Sigma). Slopes were incubated at 30°C until the cultures had conidiated and spores were resuspended in 0.1% (v/v) Tween 20 (Sigma). Liquid batch cultures were inoculated with 3 × 10^5 ^spores/ml, grown for 44 h in 100 ml of starch-containing ACMN/N/P medium [53] in 250 ml conical flasks at 25°C and shaken at 150 r.p.m. Batch AB4.1 cultures were treated with ER stress reagent (DTT or tunicamycin) for 2 h. DTT stock was prepared in water and tunicamycin stock in DMSO. DTT or tunicamycin were added to the liquid medium at a final concentration of 20 mM or 10 μg/ml, respectively. Control cultures had an equivalent volume of sterile water or DMSO added. For tunicamycin treatment with germlings of *A. niger*, fresh spore cultures were prepared as previously described and incubated for 5 h. A sample was taken for microscopic examination of germination, which was in excess of 90%. Cultures were treated by tunicamycin (10 μg/ml) or DMSO for 1 h, after which the germlings were harvested by filtration and frozen in liquid nitrogen. Continuous cultures were carried out in an Applikon (FT Applikon Ltd., Tewkesbury, UK) bioreactor (2.1 l full working volume) according to published methods [54]. The medium (one tenth strength SCM contained 5 gl^-1 ^maltose as the carbon source. Cultures were inoculated with 5 × 40 ml shake flask pre-cultures, which had been growing in medium containing 10 gl^-1 ^of maltose for approximately 24 hours. Cultures were maintained at 30°C ± 1°C and pH 5.5 ± 0.1, agitated at 1200 rpm with a 3 six-bladed (48 mm diameter) Rushton turbine impeller and aerated with 0.7 vvm (l air [l culture]^-1^min^-1^). Foaming was controlled by continuous addition of a mixture of polypropylene glycol (PPG) of different molecular weights PPG 1025 (BDH): PPG 2025 (BDH): FoamMaster PPG (mixed molecular weight; Henkel Performance Chemicals, Leeds, UK) in the ratio 2:2:1 [15] to give a final concentration of approximately 0.01% (v/v) PPG. The dilution rate was kept constant at 0.080 ± 0.006 h^-1^. In order to reduce the degree of attachment of biomass to the surfaces inside the bioreactor, the impeller speed was increased to 1500 rpm once a day for about 15 minutes after sampling. The maximum specific growth rate (μ_max_) during the batch growth phase in bioreactors was estimated from the CO_2 _output using an ADC 7000 infrared Gas Analyzer (The Analytical Development Co. Ltd.; U.K) and CO_2 _evolution rate was also measured online throughout the whole experiment to monitor steady state.

### Total RNA extraction

Total RNA was prepared from each condition according to the TRIzol reagent protocol (Invitrogen). Each sample was treated with RQ1 RNase-Free DNase (Promega) according to the manufacturer's instructions, followed by a phenol:chloroform extraction and ethanol precipitation. An additional cleanup was performed using the RNEasy Mini Kit (Qiagen), following the manufacturer's RNA Clean-up protocol.

### Polysome analysis

Ribosomal fractions were prepared according to the method described for polysome analysis [21], modified as follows. At the time of harvest, cycloheximide was added to a final concentration of 0.1 mg/ml to trap elongating ribosomes. The cultures were swirled rapidly and chilled on ice for 10 minutes. Fungal material was pelleted by centrifugation at 11000 g for 10 min at 4°C. The pellet was then resuspended in 5 ml of polysome extraction buffer (20 mM Tris-HCl pH 8.0, 140 mM KCl, 1.5 mM MgCl_2_, 1% Triton X-100, 0.1 mg/ml cycloheximide, 1.0 mg/ml heparin, 0.5 mM DTT) and sedimented. This washing step was repeated and cells were frozen in liquid nitrogen and stored at -80°C.

Approximately 0.25 g of cells was ground in liquid nitrogen with a mortar and a pestle, and the powder was resuspended in 750 μl of ice-cold polysome extraction buffer. Excess cell debris were removed by sedimentation at 4000 g for 5 min at 4°C. The supernatant was clarified by further centrifugation (12000 g, 10 min, 4°C). Each sample was loaded on an 8 ml 15 to 60% (w/v) sucrose gradient and sedimented at 150000 g (55000 rpm) and 4°C in a Beckman MLA-80 rotor for 135 min. Sucrose solutions were prepared in 50 mM Tris acetate pH 7.0, 50 mM NH_4_Cl, 12 mM MgCl_2_, 1 mM DTT. The gradient was fractionated with a density gradient fractionator (Foxy Jr. Fraction Collector, ISCO), whilst monitoring absorbance at 254 nm using a UA-6 UV detector (ISCO). 0.5 ml fractions were collected from the top of the gradient directly into 1 ml volume of 6 M guanidine hydrochloride. RNA was precipitated by adding an equal volume of 100% ethanol and resuspended in Tris-EDTA (TE, pH 8.0). The RNA was again precipitated by addition of 50 μl of 3 M sodium acetate (pH 5.2) and 1 ml of 100% ethanol, and resuspended in TE (pH 8.0).

For RNAs destined for microarray analyses, the polysomal fractions and non-polysomal fractions were pooled respectively. Each fraction was treated with RQ1 RNase-Free DNase (Promega) and was again cleaned up by applying the samples to an RNeasy mini column (Qiagen).

### Northern analysis and RT-PCR amplification analyses

Northern blot hybridization was performed using the formaldehyde-based system NorthernMax as described by manufacturer and transfer was achieved to BrightStar-Plus membranes (Ambion Inc., Austin, Tex.). [α-32P]dUTP-labeled antisense RNA probes were prepared with the Lig'nScribe and the Strip-EZ RNA kits according to the manufacturer's instructions (Ambion Inc., Austin, Tex.). Coding sequences for differentially expressed genes were downloaded from the DSM *A. niger *database [13] and PCR primers for each selected gene were designed with Primers 3.0 (Whitehead Institute, Massachusetts Institute of Technology, Boston, MA). The PCR primers used to generate probes for Northern blotting and for RT-PCR (including for RT-PCR across the hacA mRNA intron) are listed in Table 4. Probes were generated from genomic DNA of *A. niger *strains by PCR amplification. The PCR conditions were as follows: an initial denaturation of 94°C for 3 min was followed by 30 cycles of 94°C for 30 s, 57°C for 1 min and 72°C for 1 min. For densitometric analysis, signals were quantified with a Fuji film BAS2000 phosphorimaging system.

Reverse transcriptions were carried out using the SuperScript II Reverse Transcriptase system (Invitrogen, Life Technologies) according to the manufacturer's instructions.

### Genome sequence and microarray analysis

Processing of mRNA to cDNA, labelling, hybridization to *A. niger *Affymetrix GeneChips (Affymetrix, Inc., Santa Clara, CA) and fluorescence scanning were performed at the Nottingham Arabidopsis Stock Center (NASC, Loughborough, UK) Affymetrix facility. The Affymetrix Microarray Suite version 5.0 program was used to normalize microarray data and to calculate signal intensity, detection p-value, signal log ratio and change p-value using the statistical algorithms of version 5.0. The detection p-value was used to assign a call whether a measured transcript was detected at significant level as P (present), below detection level as A (absent) or in-between as M (marginal). The change p-value based on Wilcoxon's Signed Rank test of 12 probe pair comparisons, was used to provide a measure of the likelihood of change between two GeneChips and direction, assigning a change call as I (induced), D (decreased) or NC (not changed). The signal and signal log ratio were computed by taking probe pair intensities (each transcript is represented by 12 probe pairs) of one GeneChip or across two GeneChips, respectively. For each treatment condition, for all 14555 probe sets, the calculated signal log ratios (in base 2) were converted into fold change of mRNA level relative to untreated or control cultures, to make interpretation more intuitive.

In total, 12 Affymetrix Genechips measurements were obtained with total RNA samples from which 6 duplicated comparisons between the treated samples and the respective control were analysed: 2 for the DTT treatment, 2 for the tunicamycin treatment and 2 for the t-PA production. Genes were considered to be differentially expressed specifically by each treatment if (i) expression levels changed by at least 1.5 fold in both independent replicates, (ii) the mRNAs were assigned at least 2× p-values over the 4 detection calls, and (iii) the change call in gene expression was in the same direction (increased or decreased) in duplicated experiments. Additionally, a total of 8 Affymetrix Genechips were hybridized with polysomal and non-polysomal samples treated or not by DTT, each conditions being analysed twice. The Spotfire program was used to determine the list of genes meeting these restriction criteria and to determine the overlap in the gene sets. Finally, manual inspection was performed for each selected entry group to remove repeated or control entries. The Spotfire Decision Site for Functional Genomics was used to perform hierarchical clustering (Euclidean distance, UPGMA) and to group records according to their similarity in a dendrogram.

The *A. niger *genome and gene sequence can be accessed through [55].

## Authors' contributions

This study was carried out predominantly by TG in the laboratory of DA. TG was funded by DSM Food Specialities and NvP was directly involved in the analysis of Affymetrix GeneChip data. Detailed discussions on this analysis were held at DSM and involved HS, NvP, TG and DA. KL and GR provided samples of *A. niger *biomass producing tPA. TGoosen and CvdH collaborated by undertaking some stress studies. All authors contributed in discussions of the approach and interpretation of results.

## Supplementary Material

Additional file 1 Scaling (normalization) factors for the GeneChips used in the stress studies. The figure provided show that overall signal intensities were comparable throughout all GeneChips. Click here for file

Additional file 2 Overall variations in gene expression levels induced by tunicamycin treatment. In panel A, signals of tunicamycin treatment (y-axis) vs DMSO (x-axis). In panel B, distribution of signal log ratio's (^2 ^log). Click here for file

Additional file 3 Overall variations in gene expression levels induced by t-PA expression. In panel A, signals of t-PA treatment (y-axis) vs control strain (x-axis). In panel B, distribution of signal log ratio's (^2^log). Click here for file

Additional file 4 Overall variations in gene expression levels induced by DTT treatment. In panel A, signals of DTT (y-axis) vs control strain (x-axis). In panel B, distribution of signal log ratio's (^2 ^log). Click here for file

Additional file 5 Genes induced by tPA and/or tunicamycin treatments. The fold changes in expression are indicated for all the treatments (Tun = tunicamycin, DTT = dithiothreitol, tPA = production of t-PA). Similarities are expressed in comparison with the *Saccharomyces cerevisiae *genome except when indicated. The symbol* indicates that the value do not meet the defined restrictive criteria. The genes induced by the three treatments are marked in bold. Click here for file

Additional file 6 Genes repressed by tPA and/or tunicamycin treatments. The fold changes in expression are indicated for all the treatments (Tun = tunicamycin, DTT = dithiothreitol, tPA = production of t-PA). Similarities are expressed in comparison with the *Saccharomyces cerevisiae *genome except when indicated. The symbol* indicates that the value do not meet the defined restrictive criteria. Click here for file

## Figures and Tables

**Table 1:** Genes induced by at least 2 treatments.

DSM code	Predicted protein				
**Protein Folding**		**Tun 1 h**	**Tun 2 h**	**DTT**	**tPA**

**An01g13220**	**similar to the chaperone LHS1**	**3.4 **± 0.5	**1.5 **± 0.7	**2.5 **± 0.3	**1.7 **± 0.8
**An02g14800**	**Protein disulfide isomerase PDIA**	**1.7 **± 0.1	**2.6 **± 0.1	**2.2 **± 0.004	**1.9 **± 0.01
**An01g04600**	**Protein disulfide isomerase PRPA**	**3.8 **± 0.2	**2.6 **± 0.3	**4.0 **± 0.3	**2.2 **± 0.2
**An01g08420**	**calnexin CNXA**	**3.0 **± 0.3	**2.3 **± 0.1	**3.2 **± 0.5	**2.2 **± 0.1
**An11g04180**	**chaperone BIPA **	**3.2 **± 0.7	**2.6 **± 0.6	**4.7 **± 0.6	**2.4 **± 0.008
**An16g07620**	**similar to ER oxidising protein ERO1**	**1.9 **± 0.7	**2.6 **± 0.4	**3.1 **± 0.9	**3.1 **± 0.5
An18g02020	Protein disulfide isomerase TIGA	*1.3 ± 0.5	1.7 ± 0.4	*1.7 ± 0.03	1.8 ± 0.02
An11g11250	similar to the chaperone P58IPK *Homo sapiens*	2.5 ± 0.6	1.9 ± 0.07	*1.3 ± 0.3	1.5 ± 0.05
An05g00880	similar to dnaJ protein homolog SCJ1	2.5 ± 0.3	1.5 ± 0.06	2.1 ± 0.3	*1.6 ± 0.2
**Translocation/signal peptidase complex**					

An01g13070	similar to ER protein-translocation complex subunit SEC63	*1.3 ± 0.5	1.6 ± 0.09	*1.7 ± 0.2	2.5 ± 0.1
An16g08830	similar to component of subcomplex SEC71	1.8 ± 0.2	*1.4 ± 0.08	1.9 ± 0.4	*1.7 ± 0.04
An17g00090	similar to translocation complex subunit SEC72	1.7 ± 0.2	1.6 ± 0.2	2.1 ± 0.5	*1.3 ± 0.1
An01g11630	similar to translocation complex component SSS1	2.5 ± 0.2	1.6 ± 0.3	1.6 ± 0.04	*1.6 ± 0.1
**An09g05420**	**similar to signal peptidase subunit SPC3**	**2.7 **± 0.3	**1.8 **± 0.2	**1.5 **± 0.2	**1.8 **± 0.8
An01g00560	similar to signal peptidase subunit SEC11	2.2 ± 0.4	1.9 ± 0.06	*1.1 ± 0.2	1.7 ± 0.009
An15g06470	similar to signal sequence receptor α-subunit	1.8 ± 0.6	1.5 ± 0.07	*1.5 ± 0.4	1.5 ± 0.08
**Glycosylation**					

An14g05910	similar to mannosyltransferase ALG2	2.3 ± 0.5	*2.9 ± 1.1	*1.7 ± 0.2	1.5 ± 0.1
An18g02360	similar to mannosyltransferase ALG3	2.4 ± 0.4	4.8 ± 2.1	*-1.4 ± 0.3	2.0 ± 0.3
**An03g04410**	**similar to glucosyltransferase ALG5 **	**1.8 **± 0.3	*1.7 ± 0.001	**2.2 **± 0.4	**1.7 **± 0.1
An02g03240	similar to N-acetylglucosaminephosphotransferase ALG7	1.8 ± 0.3	2.8 ± 0.5	2.1 ± 0.1	*1.7 ± 0.2
An07g04190	similar to glycosyltransferase WBP1	1.9 ± 0.2	1.8 ± 0.09	*1.4 ± 0.2	1.7 ± 0.09
An02g14560	oligosaccharyltransferase alpha subunit OSTA	1.7 ± 0.09	1.6 ± 0.06	*-1.4 ± 0.007	1.6 ± 0.4
An18g03920	similar to oligosaccharyltransferase subunit OST2	1.9 ± 0.4	1.5 ± 0.2	1.5 ± 0.1	*1.2 ± 0.2
**An18g04260**	**similar to UDP-galactose transporter HUT1**	**2.3 **± 0.2	*-1.0 ± 0.5	**2.1 **± 0.3	**1.9 **± 0.2
An18g06220	similar to alpha-mannosidase MNS1	*1.1 ± 0.5	2.4 ± 0.6	*1.2 ± 0.004	1.8 ± 0.1
An13g00620	similar to beta subunit of an ER alpha-glucosidase	*1.4 ± 0.5	1.9 ± 0.2	*-1.8 ± 0.1	1.7 ± 0.2
An15g01420	similar to glucosidase I CWH41	*1.3 ± 0.5	1.7 ± 0.5	*-1.8 ± 0.2	1.6 ± 0.01
An04g05250	similar to dolichol synthesis protein RER2	1.7 ± 0.1	*1.3 ± 0.08	2.2 ± 0.1	*1.2 ± 0.1
An02g14940	similar to flippase RFT1	*1.5 ± 0.5	*-1.0 ± 0.9	1.7 ± 0.1	1.7 ± 0.1
**Vesicle trafficking/transport**					

**An03g04940**	**similar to COPII vesicle coat component protein ERV41**	**2.2 **± 0.07	**1.9 **± 0.08	**2.4 **± 0.8	**2.3 **± 0.6
An01g04320	similar to COPII vesicle coat component protein ERV46	2.1 ± 0.2	2.4 ± 0.3	*1.5 ± 0.1	2.1 ± 0.05
An02g04250	similar to ER protein P58 (lectin family) *Rattus norvegicus*	1.7 ± 0.5	1.8 ± 0.2	*1.2 ± 0.01	1.5 ± 0.3
An02g08450	secretory gene product NSFA	1.5 ± 0.2	*1.2 ± 0.006	2.5 ± 0.06	*1.1 ± 0.06
An08g06780	similar to ER to Golgi transport protein USO1	2.0 ± 0.1	*6.5 ± 3.2	5.3 ± 3.6	*1.7 ± 0.6
**Proteolytic degradation**					

An16g06750	similar to D-stereospecific aminopeptidase	*2.0 ± 0.5	2.1 ± 0.1	14.0 ± 8.0	*2.1 ± 0.2
An08g09000	similar to ubiquitin like protein DSK2	1.7 ± 0.4	*1.0 ± 0.1	3.0 ± 0.2	*-1.1 ± 0.2
An09g00950	similar to aminopeptidase DAP *Ochrobactrum anthropi*	1.6 ± 0.2	*-1.7 ± 0.3	6.2 ± 1.4	*1.1 ± 0.2
**Lipid/Inositol metabolism**					

**An02g13410**	**similar to acetyl-coenzyme A transporter AT-1**	**2.4 **± 0.04	**1.8 **± 0.1	**5.4 **± 0.3	**2.3 **± 0.2
An11g02990	similar to cytochrome P450 DIT2	*-1.2 ± 0.5	*1.2 ± 0.002	2.0 ± 0.3	1.6 ± 0.03
An14g07030	similar to carboxylesterase *Aspergillus fumigatus*	*1.6 ± 0.5	*-1.9 ± 1.2	43.0 ± 5.3	1.7 ± 0.2
**Cellular transport**					

An12g03150	similar to multidrug resistance protein ATRD	2.0 ± 0.4	*-1.1 ± 0.3	10.1 ± 2.5	*-1.1 ± 0.1
**Translation**					

An14g06610	similar to elongation factor Eef1 alpha-A chain TEF2	1.6 ± 0.1	*1.6 ± 0.3	8.6 ± 1.3	*1.4 ± 0.3
**Stress related**					

An12g03580	similar to glutathione S-transferase 3 MGST3 *H. sapiens*	*1.2 ± 0.5	*1.3 ± 0.1	3.2 ± 0.5	5.1 ± 1.4
An01g14100	weakly similar to stress protein HERP Mus musculus	2.0 ± 0.1	*1.5 ± 0.2	1.5 ± 0.3	*1.8 ± 0.3
**Cell Cycle and DNA processing**					

An01g08170	similar to DNA repair endonuclease RAD1 *S. pombe*	1.8 ± 0.5	*1.8 ± 0.2	2.2 ± 1.1	*1.6 ± 0.2
**C-compound and carbohydrate metabolism**					

An09g06400	similar to chitinase CHIA *Aspergillus nidulans*	-2.1 ± 0.5	*1.0 ± 0.008	2.4 ± 0.3	2.3 ± 0.8
An16g09070	similar to glucosamine-6-phosphate deaminase *A. fumigatus*	*-1.2 ± 0.5	*1.4 ± 0.2	2.1 ± 0.08	2.4 ± 0.7
**Unclassified**					

**An08g03960**	**hypothetical endoplasmic reticulum associated protein**	**2.1 **± 0.09	**1.7 **± 0.07	**3.3 **± 1.0	**1.6 **± 0.06
An02g00120	hypothetical protein	*2.4 ± 0.5	*1.2 ± 0.3	2.0 ± 0.5	1.8 ± 0.07
An08g03970	hypothetical protein	*1.7 ± 0.5	2.0 ± 0.4	*1.2 ± 0.02	1.8 ± 0.1
An09g00650	hypothetical protein	2.0 ± 0.3	*-1.30.4	2.7 ± 0.2	4.0 ± 1.4
An17g00660	hypothetical protein	*-1.1 ± 0.2	*-1.5 ± 0.6	5.0 ± 2.7	1.6 ± 0.1
An11g06970	hypothetical protein	*3.1 ± 0.3	*1.7 ± 0.7	17.3 ± 6.0	2.3 ± 0.6
An16g00070	hypothetical protein	*-1.4 ± 0.5	*1.1 ± 0.1	5.1 ± 0.1	1.5 ± 0.2
An16g08470	hypothetical protein	*1.8 ± 0.3	*1.1 ± 0.2	1.5 ± 0.1	1.5 ± 0.05
An02g14500	hypothetical protein	*-1.2 ± 0.4	*1.0 ± 0.1	9.3 ± 2.7	1.7 ± 0.1
An03g00720	hypothetical protein	*1.1 ± 0.5	*-1.2 ± 0.06	2.6 ± 1.1	1.9 ± 0.7
An04g02250	questionable ORF	*2.2 ± 0.3	2.0 ± 0.2	20.2 ± 10.3	*1.3 ± 0.006
An14g01990	hypothetical protein	3.0 ± 0.4	*-1.4 ± 0.2	4.3 ± 0.9	*-1.1 ± 0.03
An08g04260	hypothetical protein	2.0 ± 0.6	*1.7 ± 0.3	2.4 ± 0.01	*1.3 ± 0.07
An07g10280	hypothetical protein	2.0 ± 0.7	*1.6 ± 0.08	1.8 ± 0.9	*1.6 ± 0.2
An09g06130	hypothetical protein	2.0 ± 0.3	*2 ± 0.2	1.6 ± 0.4	*1.4 ± 0.09
An08g09860	hypothetical protein	2.3 ± 0.6	*1.2 ± 0.4	7.4 ± 1.0	*-1.4 ± 0.1
An18g06120	similar to secreted protein vc33_1	1.9 ± 0.2	*-1.1 ± 0.1	2.2 ± 0.1	*1.0 ± 0.05
An14g06550	hypothetical protein	2.1 ± 0.3	*1.2 ± 0.1	2.8 ± 0.6	*1.2 ± 0.1
An15g02650	hypothetical protein	2.8 ± 1.5	*1.1 ± 0.2	2.8 ± 0.6	*1.1 ± 0.1
An11g10800	similar to probable isochorismatase	2.3 ± 0.3	*1.0 ± 0.002	11.5 ± 2.2	*1.2 ± 0.2
An18g01690	hypothetical protein	2.3 ± 1.5	*2.1 ± 0.5	2.4 ± 0.9	*1.1 ± 0.1
An16g08680	hypothetical protein	1.7 ± 0.4	*-1.1 ± 0.2	6.7 ± 0.5	*1.1 ± 0.07

The fold changes in expression are indicated for all the treatments (Tun = tunicamycin, DTT = dithiothreitol, tPA = production of t-PA) and are averaged over two experiments. Similarities are expressed in comparison with the *Saccharomyces cerevisiae *genome except when indicated. The symbol* indicates that the value does not meet one of the 3 restrictive criteria defined in the Method section. The genes induced by the three treatments are marked in bold.

**Table 2:** Genes repressed by at least 2 treatments.

DSM code	Predicted protein				
**Protein Fate**		**Tun 1 h**	**Tun 2 h**	**DTT**	**tPA**

*Proteolytic Degradation*					
An12g05960	similar to dipeptidyl peptidase II DPPII *Rattus norvegicus*	*1.3 ± 0.5	*1.6 ± 0.2	-1.9 ± 0.3	-1.5 ± 0.6
An03g01660	similar to vacuolar aminopeptidase Y APE3	-2.1 ± 0.3	*1.0 ± 0.05	-1.7 ± 0.7	*1.1 ± 0.02
An03g05200	similar to carboxypeptidase S1 *Penicillium janthinellum*	-2.1 ± 0.4	*1.1 ± 0.4	-1.8 ± 0.6	*-1.4 ± 0.08
An04g01440	similar to vacuolar aspartyl protease PEP4	-2.1 ± 0.3	*1.1 ± 0.2	-1.6 ± 0.04	*-1.3 ± 0.03
An08g08750	carboxypeptidase Y CPY	-1.9 ± 0.2	*1.0 ± 0.06	-2.2 ± 0.02	*-1.3 ± 0.04
An14g00620	similar to aminopeptidase *Aspergillus oryzae*	-2.4 ± 0.5	*-1.0 ± 0.01	-2.3 ± 0.1	*-1.1 ± 0.2
**Lipid metabolism**					

An16g01880	similar to lysophospholipase *Aspaergillus foetidus*	*-2.2 ± 0.5	-1.7 ± 0.3	-99.8 ± 5.6	1.4 ± 0.8
An02g09540	similar to choline permease HNM1	*1.1 ± 0.5	*1.0 ± 0.6	-2.4 ± 0.9	-1.9 ± 0.1
An16g06090	similar to choline permease HNM1	*-1.3 ± 0.3	*1.1 ± 0.01	-1.9 ± 0.2	-2.2 ± 0.5
An15g06810	similar to cytochrome-b5 reductase MCR1	*1.1 ± 0.2	*1.6 ± 0.1	-1.7 ± 0.3	-2.6 ± 011
An01g03350	similar to C-8 sterol isomerase ERG1 *Neurospora crassa*	-1.9 ± 0.3	*1.2 ± 0.2	-2.1 ± 0.6	*-1.2 ± 0.3
An02g03580	similar to lipid metabolism protein ERG28	-1.8 ± 0.2	*1.2 ± 0.02	-2.0 ± 0.2	*-1.3 ± 0.06
An01g07000	similar to C-14 sterol reductase ERG24	-1.7 ± 0.1	*1.2 ± 0.2	*3.9 ± 0.4	-1.9 ± 0.08
**Cell wall**					

An16g03370	similar to protein involved in of cell wall biogenesis CWH43	-2.0 ± 0.2	*1.2 ± 0.1	-2.4 ± 0.1	*-1.2 ± 0.01
An16g07040	similar to cell wall glucanase SCW10	-1.7 ± 0.1	*-1.1 ± 0.07	-4.0 ± 0.5	*-1.0 ± 0.04
**Amino acid metabolism**					

An04g00990	NADP-dependent glutamate dehydrogenase GDHA	*1.1 ± 0.3	*-3.5 ± 2.0	-3.6 ± 0.7	-1.5 ± 0.005
An16g01610	similar to hydroxylase BBH *Rattus norvegicus*	*-1.2 ± 0.5	-2.5 ± 0.7	-13.5 ± 0.1	*-1.0 ± 0.5
An01g14730	similar to lactonizing enzyme I TcMLE *Trichosporon cutaneum*	-1.6 ± 0.1	*-1.3 ± 0.1	-1.5 ± 0.2	*1.3 ± 0.2
An02g00190	similar to enantiomer-selective amidase AMDA *Rhodococcus sp*.	-2.9 ± 0.5	*1.1 ± 0.2	-2.3 ± 0.3	*-1.3 ± 0.5
An12g10000	similar to GABA permease GABA *Aspergillus nidulans*	*-1.0 ± 0.08	*1.0 ± 0.1	-5.5 ± 1.9	-1.8 ± 0.09
**Phosphate Metabolism**					

An12g01910	similar to phytase PHYA3 *Aspergillus fumigatus*	-1.5 ± 0.1	-1.6 ± 0.3	-21.1 ± 2.5	*-1.0 ± 0.1
**C-compound and carbohydrate metabolism**					

An09g00270	alpha-galactosidase AGLC	*-1.2 ± 0.1	-2.0 ± 0.5	-5.9 ± 2.0	*1.1 ± 0.3
An09g00260	alpha-galactosidase AGLC	*-2.6 ± 0.5	-2.1 ± 0.3	-6.0 ± 4.3	*1.1 ± 0.2
An01g01540	similar to alpha.alpha-trehalase TREA *A. nidulans*	-3.0 ± 0.6	*-1.1 ± 0.1	-2.7 ± 0.6	*1.2 ± 0.02
An01g00780	endo-1.4-xylanase XYNB	-3.4 ± 0.6	*1.5 ± 0.4	-4.3 ± 0.9	*1.0 ± 0.1
An05g00930	similar to mitochondrial malic enzyme MAE1	-2.1 ± 0.2	*1.2 ± 0.1	-4.0 ± 1.0	*-1.0 ± 0.04
An19g00090	similar to exo-beta-1.3-glucanase CMG1 *Coniothyrium minitans*	-2.6 ± 0.3	*1.6 ± 0.1	-4.6 ± 1.0	*3.7 ± 0.06
An11g04780	similar to protein involved in active glycerol uptake GUP1	-1.7 ± 0.2	*1.2 ± 0.2	-2.4 ± 0.3	*1.0 ± 0.1
**metabolism of vitamins. cofactors. and prosthetic groups**					

An01g04250	similar to uroporphyrinogen decarboxylase HEM12	*-1.2 ± 0.06	*1.1 ± 0.04	-1.6 ± 0.1	-1.6 ± 0.1
**Cellular transport**					

An12g10320	similar to high-affinity zinc transport protein ZRT1	*1.1 ± 0.05	*1.4 ± 0.04	-2.2 ± 0.04	-3.2 ± 1.1
An16g06740	similar to cadmium resistance protein YCF1	*-1.4 ± 0.2	*-1.7 ± 0.5	-1.7 ± 0.2	-1.7 ± 0.3
**Cell Rescue. Defense and Virulence**					

An07g00570	similar to membrane protein PTH11 *Magnaporthe grisea*	*-1.1 ± 0.1	*-1.0 ± 0.04	-2.7 ± 0.06	-2.0 ± 0.1
An18g00980	similar to membrane protein PTH11 *M. grisea*	*-3.0 ± 0.5	*1.4 ± 0.2	-4.6 ± 0.2	-2.0 ± 0.1
An16g05920	similar to membrane protein PTH11 *M. grisea*	*1.1 ± 0.2	-3.9 ± 1.1	-5.5 ± 1.1	*1.0 ± 0.08
**Nucleotide/nitrogen and sulfur metabolism**					

An10g00800	similar to purine nucleoside permease NUP *Candida albicans*	-5.4 ± 0.8	*-1.5 ± 0.07	-31.9 ± 2.7	*1.2 ± 0.1
An18g01220	similar to allantoate permease DAL5	-1.6 ± 0.1	*1.6 ± 0.2	-4.2 ± 0.06	*-1.2 ± 0.05
**Other**					

An07g06530	similar to multicopy suppressor SUR7	*2.0 ± 0.4	*1.7 ± 0.2	-5.0 ± 1.9	-1.6 ± 0.1
An16g05910	similar to putative cytochrome P450 *A. fumigatus*	*-1.2 ± 0.08	-2.3 ± 0.5	-5.8 ± 0.2	*2.2 ± 0.05
An11g09870	similar to palmitoleyltransferase ERF2	-1.6 ± 0.1	*1.4 ± 0.2	-2.1 ± 0.3	*1.0 ± 0.01
**Unclassified**					

An02g03570	similar to membrane protein YBR159w	-2.1 ± 0.2	*-1.3 ± 0.3	*-1.9 ± 1.0	-1.6 ± 0.2
An11g07340	similar to hypothetical O-methyl transferase ENCK	*1.3 ± 0.1	*1.3 ± 0.5	-4.2 ± 0.6	-4.2 ± 1.7
An12g09640	similar to hypothetical GTP cyclohydrolase	*2.6 ± 0.5	*1.3 ± 0.4	-3.3 ± 0.2	-1.9 ± 0.5
An01g09220	weakly similar to tyrosinase MELC2	*-2.1 ± 0.5	*2.1 ± 0.2	-5.2 ± 1.7	-1.8 ± 0.4
An03g00770	similar to allergic allergen rAsp f 4 *A. fumigatus*	*-2.3 ± 0.6	*-1.1 ± 0.3	-2.6 ± 0.1	-2.8 ± 0.6
An08g03760	hypothetical protein	*-1.1 ± 0.1	*-1.3 ± 0.07	-4.0 ± 1.8	-2.4 ± 0.3
An02g08330	hypothetical protein	*1.2 ± 0.2	*-17 ± 11.3	13.5 ± 5.2	-1.8 ± 0.1
An06g01000	hypothetical protein	*-1.1 ± 0.1	*-1.0 ± 0.05	-1.83 ± 0.5	-2.3 ± 0.1
An11g07020	hypothetical protein	*1.3 ± 0.3	*1.2 ± 0.02	-2.1 ± 0.3	-1.8 ± 0.2
An02g08300	hypothetical protein	*2.2 ± 0.5	*-1.7 ± 0.3	-3.2 ± 0.8	-8.6 ± 0.9
An03g00840	hypothetical protein	1.8 ± 0.2	*-1.2 ± 0.2	*1.3 ± 0.1	-2.4 ± 0.9
An18g01000	hypothetical protein	*-2.4 ± 0.5	*1.1 ± 0.1	-4.6 ± 0.5	-1.8 ± 0.2
An13g01520	hypothetical protein	-1.5 ± 0.1	*-2.9 ± 0.2	*-1.0 ± 0.4	-1.9 ± 0.2
An02g00120	hypothetical protein	-2.4 ± 0.5	*1.2 ± 0.3	-2.0 ± 0.5	*-1.8 ± 0.07
An04g01690	hypothetical protein	-2.1 ± 0.4	*-1.0 ± 0.3	-3.1 ± 1.1	*-1.1 ± 0.03
An05g01770	hypothetical protein	-1.5 ± 0.1	*-1.5 ± 0.3	-5.8 ± 1.0	*1.0 ± 0.5
An07g05160	hypothetical protein	-1.7 ± 0.1	*-1.2 ± 0.2	-2.1 ± 0.05	*1.0 ± 0.08
An07g08400	strong similarity to allergen rAsp f 4 *A. fumigatus*	-1.8 ± 0.2	*1.3 ± 0.2	-4.2 ± 1.2	*1.1 ± 0.07
An08g08600	similar to RTN2	-1.9 ± 0.2	*-1.0 ± 0.01	-3.3 ± 0.6	*-1.0 ± 0.1
An12g10590	hypothetical protein	-2.6 ± 0.4	*1.1 ± 0.03	-12.0 ± 2.1	*1.0 ± 0.02
An15g01740	similar to ER protein of unknown function	-1.6 ± 0.1	*1.4 ± 0.1	-2.1 ± 0.3	*1.0 ± 0.1

The fold changes in expression are indicated for all the treatments (Tun = tunicamycin, DTT = dithiothreitol, tPA = production of t-PA) and are averaged over two experiments. Similarities are expressed in comparison with the *Saccharomyces cerevisiae *genome except when indicated. The symbol* indicates that the value does not meet one of the 3 restrictive criteria defined in the Method section.

**Table 3:** Examples of genes for which DTT treatment resulted in > 2 fold shift of transcripts from the non-polysomal to polysomal samples, or *vice versa*, relative to untreated controls.

**DSM code**	**Predicted protein**	**Polysomal/non-polysomal Control**	**Polysomal/non-polysomal DTT treated**	**DTT induced shift**
**From non-polysomal to polysomal fractions**				
An09g05420	similar to signal peptidase subunit SPC3	4.61	0.64	7.20
An15g01130	similar to brefeldin A resistance protein BFR1 (*S. pombe*)	0.79	0,26	3.04
An01g14260	similar to delta subunit of the coatomer delta-coat protein COPD (*Bos. taurus*)	1.79	0.68	2.63
An17g00270	similar to 26S ATP/ubiquitin-dependent proteinase chain S4 (*S. pombe*)	3.13	1.28	2.45
**From polysomal to non-polysomal fractions**				
**Translation**				
An02g13840	similar to cytoplasmic ribosomal protein of the small subunit S9 (*H. sapiens*)	0.47	27.28	57.91
An18g05810	similarity to cytoplasmic ribosomal protein of the small subunit S26 (*H. sapiens*)	0.51	11.22	22,03
An08g00970	ribosomal protein of the small subunit rps28	0.71	3.48	4.91
An11g09570	similar to cytoplasmic ribosomal protein of the large subunit L37.b	0.57	8.33	14.62
An12g04860	similar to cytoplasmic ribosomal protein of the large subunit L30	0.37	4.18	11.42
An01g02880	similar to cytoplasmic ubiquitin/ribosomal fusion protein CEP52	0.67	4.61	6.92
An18g04570	similar to cleavage/polyadenylation factor IA subunit CLP1	0.71	1.56	2.20
An02g09260	similar to nucleolar protein NOP5	2.12	9.22	4.34
An18g04840	similar to elongation factor 1 alpha (*Podospora anserina*)	1.88	6.02	3.21
An15g00750	Similar to elongation factor 1-gamma 1 TEF3	2.16	5.35	2.47
An07g02650	similar to translation elongation factor 3 YE3	2.96	23.88	8.07
**Proteolytic degradation**				
An11g01760	similar to proteasome 20S subunit PRE2	3.79	20.52	5.42
An02g07010	similar to ubiquitin UBI1 (*A. nidulans*)	1.1	7.5	7.12
**Lipid metabolism**				
An08g10110	similar to lipid transfer protein POX18 (*C. tropicalis*)	0.96	4.04	4.23
An03g06410	similar to methyl sterol oxidase ERG25	1.23	2.84	2.32
An12g01890	similar to squalene synthase ERG9 (*C. utilis*)	1.01	2.21	2.18
An02g10350	similar to farnesyl-pyrophosphate synthase FPPS	1.90	4.13	2.17
An07g09570	similar to phosphatidate cytidylyltransferase CDS1	0.84	1.73	2.06
**Cell Wall**				
An03g02400	similar to hydrophobin DEWA (*A. nidulans*)	0.51	1.59	3.10
An06g01550	similar to glucan synthase FKS (*Paracoccidioides brasiliensis*)	0.46	1.33	2.89
An11g00270	similar to lanosterol 14 alpha-demethylase CYP51 (*H. sapiens*)	0.64	1.44	2.25
An02g02360	similar to chitin synthase (*A. nidulans*)	0.43	0.96	2.24
**Secreted proteins**				
An12g01910	similar to phytase PHYA3 (*A. fumigatus*)	0.36	4.03	11.1
An03g06550	Glucoamylase GLAA	0.57	17.69	30.98
An02g13220	similar to lysophospholipase phospholipase B (*P. notatum*)	0.55	2.90	5.23
An09g00270	alpha-galactosidase AGLC	0.38	1.65	4.3
An02g01550	similar to serine protease (*Coccidioides immitis*)	1.78	6.69	3.76
An02g04690	similar to serine-type carboxypeptidase I CDPS (*A. saitoi*)	1.71	3.56	2.08

The DTT induced shift was determined by calculating (DTT treated polysomal/DTT treated non-polysomal)/(control polysomal/control non-polysomal).

**Table 4:** Primers for probe generation by PCR and for RT-PCR.

Gene	Primer pairs	Amplicon size (bp)
*bipA*^1,2^	5'-CAGAGACAGGCCACCAAGGACGCT-3' 5'-CGAACTTGGCGCGGGTGAGGGTCT-3'	445
*pdiA*^1^	5'-TATCTTTGCCGAGACCAAGG-3' 5'-AACAGGTTCCGACTTGATGC-3'	298
*pdiA*^2^	5'-TGATACCGAGTCTGAGTTA-3' 5'-CTCGATTCTGTTTGCCTAGC-3'	303
*An02g134101*^1^	5'-CGCATCCTACCCATACTCGT-3' 5'-GGAACCAGCGATTAGCAAAG-3'	280
*hacA*^2^	5'-CTTCTCCTACCCTAACTCCT-3' 5'-TCAAAGAGAGAGAGGGCAC-3'	397
*act1*^1^	5'-GCCTTCTACGTCTCCATCCA-3' 5'-GAACAGAGCCTCAGGAGCAC-3'	396
*act1*^2^	5'-TCATGATCGGTATGGGTCAG-3' 5'-ACGATGTTGCCGTACAGATC-3'	765

^1^: primers designed for blot probes

^2^: primers designed for RT-PCR
