# Supplementary material for: Genomic analysis of the secretion stress response in the enzyme-producing cell factory Aspergillus niger
Source: BMC Genomics. 2007 Jun 11;8:158. doi: 10.1186/1471-2164-8-158 (PMC1894978; doi:10.1186/1471-2164-8-158)
Supplement: Additional file 1 — Scaling (normalization) factors for the GeneChips used in the stress studies. The figure provided show that overall signal intensities were comparable throughout all GeneChips. [file 1471-2164-8-158-S1.doc]

**Additional file 4: Scaling (normalization) factors for the GeneChips used in the stress studies. Overall signal intensities were comparable throughout all GeneChips.**

| **Stress condition** | **Exp 1 (Exp / control)** | **Exp 2 (Exp / control)** |
| --- | --- | --- |
| Tunicamycin | 0.7 / 0.6 | 0.7 / 0.6 |
| t-PA expression | 0.4 / 0.6 | 0.4 / 0.4 |
| DTT | 0.5 / 0.4 | 0.6 / 0.4 |
