# Supplementary figures and images for: Genomic analysis of the secretion stress response in the enzyme-producing cell factory Aspergillus niger
Source: BMC Genomics. 2007 Jun 11;8:158. doi: 10.1186/1471-2164-8-158 (PMC1894978; doi:10.1186/1471-2164-8-158)

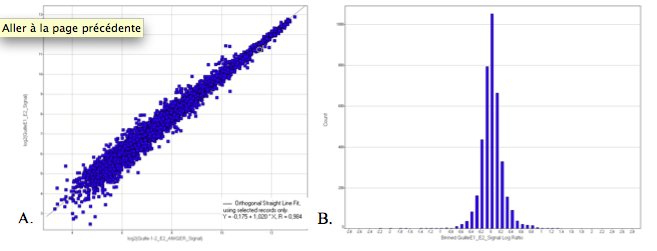

Supplement: Additional file 2 — Overall variations in gene expression levels induced by tunicamycin treatment. In panel A, signals of tunicamycin treatment (y-axis) vs DMSO (x-axis). In panel B, distribution of signal log ratio's (2 log). [file 1471-2164-8-158-S2.tiff]

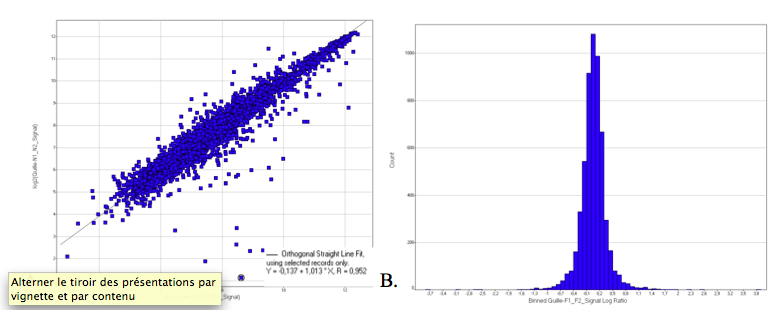

Supplement: Additional file 3 — Overall variations in gene expression levels induced by t-PA expression. In panel A, signals of t-PA treatment (y-axis) vs control strain (x-axis). In panel B, distribution of signal log ratio's (2log). [file 1471-2164-8-158-S3.tiff]

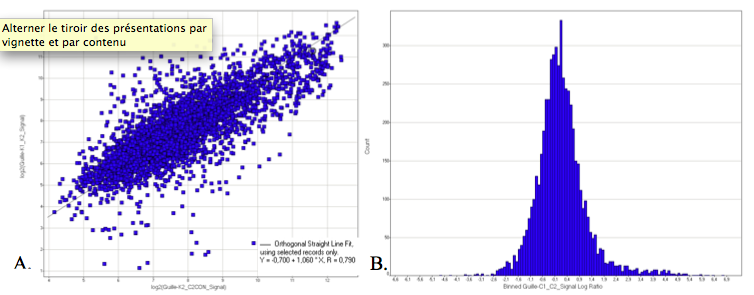

Supplement: Additional file 4 — Overall variations in gene expression levels induced by DTT treatment. In panel A, signals of DTT (y-axis) vs control strain (x-axis). In panel B, distribution of signal log ratio's (2 log). [file 1471-2164-8-158-S4.tiff]
