# Supplementary material for: Genomic analysis of the secretion stress response in the enzyme-producing cell factory Aspergillus niger
Source: BMC Genomics. 2007 Jun 11;8:158. doi: 10.1186/1471-2164-8-158 (PMC1894978; doi:10.1186/1471-2164-8-158)
Supplement: Additional file 5 — Genes induced by tPA and/or tunicamycin treatments. The fold changes in expression are indicated for all the treatments (Tun = tunicamycin, DTT = dithiothreitol, tPA = production of t-PA). Similarities are expressed in comparison with the Saccharomyces cerevisiae genome except when indicated. The symbol* indicates that the value do not meet the defined restrictive criteria. The genes induced by the three treatments are marked in bold. [file 1471-2164-8-158-S5.doc]

**Additional File 5: Genes induced by tPA and/or tunicamycin treatments.**

# DSM code Predicted protein

# Protein Folding Tun 1h Tun 2h DTT tPA

**An01g13220 similar to the chaperone LHS1 3.4 1.5 2.5 1.7**

**An02g14800 Protein disulfide isomerase PDIA 1.7 2.6 2.2 1.9**

**An01g04600 Protein disulfide isomerase PRPA 3.8 2.6 4.0 2.2**

**An01g08420 calnexin CNXA 3.0 2.3 3.2 2.2**

**An11g04180 chaperone BIPA 3.2 2.6 4.7 2.4**

**An16g07620 similar to ER oxidising protein ERO1 1.9 2.6 3.1 3.1**

An18g02020 Protein disulfide isomerase TIGA *1.3 1.7 *1.7 1.8

An04g02020 cyclophilin CYPB *-1.0 *2.5 *-2.1 1.7

An11g11250 similar to the chaperone P58IPK *Homo sapiens* 2.5 1.9 *1.3 1.5

An05g00880 similar to dnaJ protein homolog SCJ1 2.5 1.5 2.1 *1.6

# Translocation/signal peptidase complex

An03g04340 similar to ER membrane translocation facilitator SEC61 1.8 *1.6 *1.6 *1.3

An01g13070 similar to ER protein-translocation complex subunit SEC63 *1.3 1.6 *1.7 2.5

An16g08830 similar to component of subcomplex SEC71 1.8 *1.4 1.9 *1.7

An17g00090 similar to translocation complex subunit SEC72 1.7 1.6 2.1 *1.3

An01g03820 similar to ER protein-translocation complex subunit SBH2 1.6 1.8 *-4.1 *1.4

An01g11630 similar to translocation complex component SSS1 2.5 1.6 1.6 *1.6

An01g10070 similar to signal recognition particle chain SEC65 1.7 *1.2 *1.2 *1.3

An04g06890 similar to the signal recognition particle SRP72 1.5 *2.0 *1.8 *1.1

An16g07390 similar to ER signal peptidase subunit SPC2 1.9 1.5 *-1.0 *1.7

**An09g05420 similar to signal peptidase subunit SPC3 2.7 1.8 1.5 1.8**

An01g00560 similar to signal peptidase subunit SEC11 2.2 1.9 *1.1 1.7

An15g06470 similar to signal sequence receptor α-subunit 1.8 1.5 *1.5 1.5

# Glycosylation

An14g05910 similar to mannosyltransferase ALG2 2.3 *2.9 *1.7 1.5

An18g02360 similar to mannosyltransferase ALG3 2.3 4.8 *-1.4 2.0

**An03g04410 similar to glucosyltransferase ALG5 1.8 *1.7 2.2 1.7**

An02g03240 similar to N-acetylglucosaminephosphotransferase ALG7 1.8 2.8 2.05 *1.7

An07g06430 similar to glucosyltransferase KRE5 *1.3 *10.4 *1.8 2.8

An07g04190 similar to glycosyltransferase WBP1 1.9 1.8 *1.4 1.7

An02g14560 oligosaccharyltransferase alpha subunit OSTA 1.7 1.6 *-1.4 1.6

An18g03920 similar to oligosaccharyltransferase subunit OST2 1.9 1.5 1.5 *1.2

An02g14930 similar to glycotransferase gamma chain OST3 1.7 *1.4 *-1.3 *1.3

## An16g08570 similar to oligosaccharyltransferase subunit STT3 1.6 1.6 *-1.3 *1.5

**An18g04260 similar to UDP-galactose transporter HUT1 2.3 *-1.0 2.1 1.9**

An18g06220 similar to alpha-mannosidase MNS1 *1.1 2.4 *1.2 1.8

An12g00340 similar to mannosidase HTM1 *1.5 *1.2 *1.0 1.5

An09g05880 similar to alpha-glucosidase II ROT2 *1.3 1.50 *1.3 *1.5

An13g00620 similar to beta subunit of an ER alpha-glucosidase *1.4 1.9 *-1.8 1.7

An15g01420 similar to glucosidase I CWH41 *1.3 1.7 *-1.8 1.6

An16g04330 mannose phospho-dolichol synthase DPMA 1.9 1.8 *1.3 *1.5

An04g05250 similar to dolichol synthesis protein RER2 1.7 *1.3 2.2 *1.2

An01g05200 similar to dolichol synthesis protein DPM2 *Mus musculus* 2.3 *1.6 *-2 *1.2

An02g14940 similar to flippase RFT1 *1.5 *-1.0 1.7 1.7

# Vesicle trafficking/transport

**An03g04940 similar to COPII vesicle coat component protein ERV41 2.2 1.9 2.4 2.3**

An01g04320 similar to COPII vesicle coat component protein ERV46 2.1 2.4 *1.5 2.1

An02g04250 similar to ER protein P58 (lectin family) *Rattus norvegicus* 1.7 1.8 *1.2 1.5

An04g08830 similar to COP II transport vesicles protein EMP47 *-1.0 *1.3 *1.2 1.5

An02g02640 similar to Golgi membrane protein YIP1 (COPII transport) 1.7 *1.6 *1.3 *1.2

An14g00210 similar to Golgi membrane protein YIP1 (COPII transport) 1.5 *1.0 *1.0 *1.1

An02g08450 secretory gene product NSFA 1.5 *1.2 2.5 *1.1

An02g05870 similar to COP I coatomer protein SEC27 1.5 *1.3 *1.0 *1.3

An07g07340 similar to luminal ER-protein retention receptor ERD2 1.7 *1.4 *1.5 *1.3

An09g04170 similar to vesicle trafficking protein SLY1 1.7 *1.1 *1.2 *1.2

An02g11990 similar to Golgi to ER protein SEC20 2.4 *1.8 *1.5 *1.3

An04g06180 similar to exocyst subunit SEC6 1.6 *1.0 *1.1 *1.3

An08g00290 similar Golgi matrix protein RUD3 2.1 *1.0 *1.3 *1.2

An08g06780 similar to ER to Golgi transport protein USO1 2.0 *6.5 5.3 *1.7

An02g01630 similar to intra-mitochondrial sorting protein MSP1 1.8 *1.1 *1.1 *1.1

## Proteolytic degradation

An15g06280 aspartic proteinase aspergillopepsin I PEPA *-1.2 *1.1 *-1.9 1.5

An16g06750 similar to D-stereospecific aminopeptidase *2.0 2.1 14.0 *2.1

An08g09000 similar to ubiquitin like protein DSK2 1.7 *1.0 3.0 *-1.1

An01g12720 similar to ERAD protein HRD3 1.7 *1.6 *1.4 *1.4

An09g00950 similar to aminopeptidase DAP *Ochrobactrum anthropi* 1.6 *-1.7 6.2 *1.1

# Lipid/Inositol metabolism

**An02g13410 similar to acetyl-coenzyme A transporter AT-1 2.4 1.8 5.4 2.3**

An11g02990 similar to cytochrome P450 DIT2 *-1.2 *1.2 2.0 1.6

An03g01460 similar to oxidoreductase *Aspergillus fumigatus* *-1.5 *-1.6 *-1.4 24.5

An08g00560 similar to methylene-fatty acyl-phospholipid synthase OPI3 2.6 *1.1 *1.5 *1.1

An02g07610 similar to myo-inositol permease ITR2 *1.3 *-1.1 *1.1 3.2

## Cellular transport

An03g06660 similar to peptide transporter PTR2 *1.4 *-1.8 *1.3 2.5

An14g02390 similar to ammonium transport protein MEP2 *-2.3 *1.5 *1.5 2.5

An13g03990 similar to uridine permease FUI1 *-1.2 *-1.2 *1.0 2.9

An16g01820 similar to phosphate transporter PHO88 *-1.6 1.7 *1.2 *1.3

An04g04060 similar to cytochrome-c peroxidase precursor CCP1 *1.1 *-1.1 *1.2 3.2

An07g05840 similar to multidrug resistance protein HOL1 *-1.2 *-1.2 *-1.1 1.8

An05g01660 similar to pleiotropic drug resistance protein SNQ2 *-1.3 *1.4 *1.2 1.6

# An07g09840 similar to tetracyclin resistance protein TCR1 4.7 *6.5 *5.7 *2.9

An16g09180 similar to low affinity zinc transport protein ZRT2 1.8 *-1 .2 *-1.0 *1.4

An12g03150 similar to multidrug resistance protein ATRD 2.0 *-1.1 10.1 *-1.1

An19g00320 similar to vacuolar H+/Ca2+ exchanger VCX1 1.7 *1.1 *1.2 *-1.1

# Transcription

An07g03760 similar to 100 kDa coactivator SND1 *H. sapiens* *1.5 1.7 *1.5 *1.3

# Translation

An14g06610 similar to translation elongation factor Eef1 alpha-A chain TEF2 1.6 *1.6 8.6 *1.4

An04g08580 similar to protein kinase GCN2 3.1 *1.2 *1.9 *1.3

An02g14240 similar to mRNA guanylyltransferase CEG1 1.8 *1.0 *1.1 *1.1

# Stress related

An12g03580 similar to glutathione S-transferase 3 MGST3 *H. sapiens* *1.2 *1.3 3.2 5.1

# An14g07200 similar to catalase C CATC *Aspergillus nidulans* *-1.0 *1.0 *-1.7 2.0

An18g02700 similar to cytochrome P-450 monooxygenase *A. fumigatus* *-1.3 *-1.1 *-1.2 2.0

# An01g14100 weakly similar to stress protein HERP *M. musculus* 2.0 *1.5 1.5 *1.8

# Cell Cycle and DNA processing

An01g08170 similar to DNA repair endonuclease RAD1 *Schizosaccharomyces pombe* 1.8 *1.8 2.2 *1.6

# Amino acid metabolism

An01g04260 similar to cytosine deaminase FCY1 ***?** *-5.3 *1.8 2.6

An03g01590 similar to general amino acid permease GAP1 *1.0 *-2.2 *2.8 2.4

An07g05830 similar to formamidase FMDS *A.nidulans* *-1.2 *-1.2 *-7.6 2.3

An12g03460 similar to proline permease PUT4 *A.nidulans* *-1.2 *-1.1 *1.1 1.5

**C-compound and carbohydrate metabolism**

An09g06400 similar to chitinase CHIA *A.nidulans* -2.1 *1.0 2.4 2.3

An03g01550 similar to carbonylreductase *A.fumigatus* *-1.5 *-2.2 *1.3 4.7

An01g03480 similar to sorbitol dehydrogenase FUN49 ***?** *1.2 *-1.6 2.9

An17g01530 alcohol-dehydrogenase ADHA *-1.2 *-1.1 *1.1 2.8

An19g00090 similar to exo-beta-1.3-glucanase *A. fumigatus* *-2.9 *1.6 *-2.9 3.7

An03g06220 similar to glycophospholipid-anchored surface glycoprotein GAS1 *2.3 *-1.3 *1.7 4.4

An16g09070 similar to glucosamine-6-phosphate deaminase *A. fumigatus* *-1.2 *1.4 2.1 2.4

An10g00510 similar to alcohol dehydrogenase SFA1 *1.3 *-1.1 *1.2 2

An08g01740 similar to galactose –induced protein GCY1 2.0 *-1.2 *-1.3 *1.3

## Nucleotide / nitrogen and sulfur metabolism

An03g01530 similar to xanthine dehydrogenase HXA *A. nidulans* *-1.3 *1.1 *-1.4 2.2

An12g03570 orotidine-5-phosphate decarboxylase PYRA *-1.2 *-1.2 *-1.3 2.4

An12g03550 similar to allantoate permease DAL5 *1.0 *-1.2 *1.1 1.6

An06g01890 similarity to histidine triad protein HNT1 *1.1 *-1.2 *-2.3 1.5

## Other

An09g04940 similarity to transposase of TAN1 *3.1 *1.3 *10.4 5.0

An11g04750 similar to regulator of asexual and sexual reproduction DOP1 1.6 *1.1 *1.2 *1.1

An14g07030 similar to carboxylesterase *A. fumigatus* *1.6 *-1.9 43.0 1.7

An18g02690 similar to oxidase FET5 *-1.4 *-1.0 *-2.2 3.0

An13g03980 similar to oxidoreductase *A. fumigatus* *-1.4 *1.1 *-1.1 2.3

# Unclassified

**An08g03960 hypothetical endoplasmic reticulum associated protein 2.1 1.7 3.3 1.6**

An16g05020 similar to protein vip1 1.7 *1.2 *-1.2 *-1.4

An16g09020 hypothetical protein *1.0 *1.2 *1.2 1.9

An02g00120 hypothetical protein *2.4 *1.2 2.0 1.8

An16g07920 hypothetical protein *-1.1 *1.1 *-2.4 1.5

An08g03970 hypothetical protein *1.7 2.0 *1.2 1.8

**An09g00650 hypothetical protein 2.0 *-1.3 2.7 4.0**

An17g00660 hypothetical protein *-1.1 *-1.5 5.0 1.6

An11g06970 hypothetical protein *3.1 *1.7 17.3 2.3

An16g00070 hypothetical protein *-1.4 *1.1 5.1 1.5

An15g00280 hypothetical protein *2.1 *-1.1 *-1.2 2.1

An14g02470 hypothetical protein *-2.6 *-1.8 *-10.2 3.8

An02g07440 hypothetical protein *-1.1 *-1.0 *-1.1 1.6

An16g08470 hypothetical protein *1.8 *1.1 1.5 1.5

An02g14500 hypothetical protein *-1.2 *1.0 9.3 1.7

An08g08090 hypothetical protein *1.2 *-1.3 *2.5 3.4

An11g02570 hypothetical protein *1.5 *-1.1 *-1.0 1.7

An03g00720 hypothetical protein *1.1 *-1.2 2.6 1.9

An04g02250 questionable ORF *2.2 2.0 20.2 *1.3

An08g00900 hypothetical protein 1.7 *1.1 *-1.1 *1.1

An05g00110 hypothetical protein 1.7 *1.0 *-1.2 *-1.0

An05g02250 similar to hypothetical serine-threonine rich protein 1.7 *1.1 *1.2 *1.0

An14g01990 hypothetical protein 3.0 *-1.4 4.3 *-1.1

An03g03020 similar to hypothetical impala transposase 2.6 *-1.3 *-1.4 *-1.2

An08g04260 hypothetical protein 2.0 *1.7 2.4 *1.3

An07g10280 hypothetical protein 2.0 *1.6 1.8 *1.6

An09g06130 hypothetical protein 2.0 *2 1.6 *1.4

An08g03970 hypothetical protein 1.9 *2.0 *1.2 *1.8

An15g01680 similar to signal peptide-containing protein SIGP 1.7 *1.3 *1.1 *1.2

An08g09860 hypothetical protein 2.3 *1.2 7.4 *-1.4

An09g03380 hypothetical protein 1.8 *1.9 *1.1 *1.0

An18g06120 similar to secreted protein vc33_1 1.9 *-1.1 2.2 *1.0

An14g06550 hypothetical protein 2.1 *1.2 2.8 *1.2

An18g06740 hypothetical protein 1.7 *1.3 *1.0 *1.3

An12g00330 hypothetical protein 2.7 *1.9 *1.4 *1.0

An15g02650 hypothetical protein 2.8 *1.1 2.8 *1.1

An11g10800 similar to probable isochorismatase 2.3 *1.0 11.5 *1.2

An18g01690 hypothetical protein 2.3 *2.1 2.4 *1.1

An02g02260 similar to probable membrane protein 1.8 *1.6 *1.4 *1.4

An05g00090 hypothetical protein 1.81 *1.4 *-1.2 *1.0

An16g08680 hypothetical protein 1.7 *-1.1 6.7 *1.1

The fold changes in expression are indicated for all the treatments (Tun = tunicamycin, DTT = dithiothreitol, tPA = production of t-PA) and are averaged over two experiments. Similarities are expressed in comparison with the *Saccharomyces cerevisiae* genome except when indicated. The symbol* indicates that the value does not meet one of the 3 restrictive criteria defined in the Method section. The genes induced by the three treatments are marked in bold.
