# Supplementary material for: Genomic analysis of the secretion stress response in the enzyme-producing cell factory Aspergillus niger
Source: BMC Genomics. 2007 Jun 11;8:158. doi: 10.1186/1471-2164-8-158 (PMC1894978; doi:10.1186/1471-2164-8-158)
Supplement: Additional file 6 — Genes repressed by tPA and/or tunicamycin treatments. The fold changes in expression are indicated for all the treatments (Tun = tunicamycin, DTT = dithiothreitol, tPA = production of t-PA). Similarities are expressed in comparison with the Saccharomyces cerevisiae genome except when indicated. The symbol* indicates that the value do not meet the defined restrictive criteria. [file 1471-2164-8-158-S6.doc]

**Additional file 6 : Genes repressed by tPA and/or tunicamycin treatments.**

# DSM code Predicted protein

# Protein Fate Tun 1h Tun 2h DTT tPA

## Protein Folding

An06g01610 similar to heat shock protein HSP9 *Schizosaccharomyces pombe* *-1.3 *1.9 *1.0 -2.4

## Protein Modification

An13g02540 similar to ER-glutathione S-transferase GTT1 *-1.6 *1.2 *2.4 -1.6

An06g00370 similar to glutathione S-transferase ISOI *Rhodococcus* sp. *1.1 *-1.2 *7.5 -1.9

An02g00960 similar to kinase involved in translation regulation ECM32 NP *-1.1 *1.3 -1.5

## Proteolytic Degradation

An01g01720 similar to bleomycin hydrolase BLH1 *-1.2 *1.3 *-1.0 -3.2

An12g05960 similar to dipeptidyl peptidase II DPPII *Rattus norvegicus* *1.3 *1.6 -1.9 -1.5

An01g08470 similar to ubiquitin-specific protease UBP13 -1.5 *1.0 *-1.1 *1.1

An03g01660 similar to vacuolar aminopeptidase Y APE3 -2.1 *1.0 -1.7 *1.1

An03g05200 similar to carboxypeptidase S1 *Penicillium janthinellum* -2.1 *1.1 -1.8 *-1.4

An04g01440 similar to vacuolar aspartyl protease PEP4 -2.1 *1.1 -1.6 *-1.3

An08g08750 carboxypeptidase Y CPY -1.9 *1.0 -2.2 *-1.3

An14g00620 similar to aminopeptidase *Aspergillus oryzae* -2.4 *-1.0 -2.3 *-1.1

An17g00390 similar to aminopeptidase *A. oryzae* -2.7 *1.7 *-1.1 *-1.0

# Glycosylation

An15g04810 similar to alpha-1.3-mannosyltransferase MNT2 *-1.0 *1.5 *2.0 -2.0

#### Vesicle trafficking /transport

An09g02930 similar to Golgi to ER protein SLT1 *-2.1 *1.1 *-7.5 -2.3

An12g00040 similar to GTP-binding protein YPT31 *1.1 *1.2 *-1.2 -2.0

# Lipid metabolism

An18g01090 similar to lysophospholipase *A. oryzae* *-1.1 *-2.1 *3.2 -1.9

An16g01880 similar to lysophospholipase *Aspergillus foetidus* *-2.2 -1.7 -99.8 1.4

An09g01240 similar to phospholipase B PLB1 -1.8 *-1.4 *-1.1 *-1.2

An03g03550 similar to enoyl-CoA hydratase *Aspergillus fumigatus* *1.1 *5.0 *1.5 -1.5

An01g08150 similar to carbonyl reductase CBR *Homo sapiens* *2.2 *3.0 *7.7 -1.8

An02g09540 similar to choline permease HNM1 *1.1 *1.0 -2.4 -1.9

An16g06090 similar to choline permease HNM1 *-1.3 *1.1 -1.9 -2.2

An15g06810 similar to cytochrome-b5 reductase MCR1 *1.1 *1.6 -1.7 -2.6

An01g03350 similar to C-8 sterol isomerase ERG1 *Neurospora crassa* -1.9 *1.2 -2.1 *-1.2

An01g07000 similar to C-14 sterol reductase ERG24 -1.7 *1.2 *3.9 -1.9

An02g03580 similar to lipid metabolism protein ERG28 -1.8 *1.2 -2.0 *-1.3

An01g14320 similar to lipid phosphoinositide phosphatase SAC1 -1.6 *-1.1 *-1.8 *1.2

An06g01540 similar to serine C-palmitoyltransferase chain LCB1 -1.6 *1.3 *-1.3 *-1.1

An07g04200 similar to steryl ester hydrolase TGL1 -1.6 *1.1 *-1.9 *-1.0

An12g03960 similar to acyltransferase SLC1 -2.1 *-1.4 *1.0 *1.2

An15g07220 similar to sterol regulatory element binding protein UPC2 -1.6 *1.4 *-1.0 *1.1

# Cell wall

An13g02510 similar to putative glycosidase CRH1 *-1.0 *-1.4 *1.2 -2.5

An14g01840 similar to cell wall mannoprotein TIR3 *1.2 *1.1 *3.8 -1.5

An07g01160 similar to cell wall protein UTR2 -8.2 *-1.8 *-4.8 *-1.4

An16g03370 similar to protein involved in of cell wall biogenesis CWH43 -2.0 *1.2 -2.4 *-1.2

An16g07040 similar to cell wall glucanase SCW10 -1.7 *-1.1 -4.0 *-1.0

# Amino acid metabolism

## Amino acid Biosynthesis

An08g08840 similar to glutamate decarboxylase GAD1 *1.1 *1.6 *1.0 -2.4

An15g06700 similar to dihydroxy-acid dehydratase ILV3 *-1.2 *-1.7 *-1.1 -2.3

An14g00960 similar to cysteine synthase *A. fumigatus* *-1.2 *-3.1 *1.9 -1.8

An03g00280 similar to tyrosinase *Streptomyces antibioticus* -4.0 *-3.0 *-5.0 *-1.2

## Amino acid Degradation

An11g07320 similar to glyoxalase II GLO2 *-1.6 *1.5 *1.3 -6.7

An04g00990 NADP-dependent glutamate dehydrogenase GDHA *1.1 *-3.5 -3.6 -1.5

An16g01610 similar to hydroxylase BBH *R. norvegicus*  *-1.2 -2.5 -13.5 *-1.0

An01g14730 similar to lactonizing enzyme I TcMLE *Trichosporon cutaneum* -1.6 *-1.3 -1.5 *1.3

An02g00190 similar to enantiomer-selective amidase AMDA *Rhodococcus sp.* -2.9 *1.1 -2.3 *-1.3

An07g04300 similar to urea amidolyase DUR1.2 -1.7 *-1.8 *-1.2 *1.0

## Amino acid Transport

An09g00400 similar to dicarboxylic amino acid permease DIP5 *1.9 *3.3 *2.3 -2.7

An12g10000 similar to GABA permease GABA *Aspergillus nidulans* *-1.0 *1.0 -5.5 -1.8

An02g09790 similar to GABA permease UGA1 -2.2 *-2.2 *-1.3 *-1.2

## Other

An01g09550 similar to apoptosis induced factor *A. fumigatus* *-1.0 *1.4 *2.8 -2.1

An07g05900 similar to fructosyl amino acid oxidase FAOA *Aspergillus terreus* *1.1 *-1.2 *7.4 -1.5

#### Phosphate Metabolism

An12g01910 similar to phytase PHYA3 *A. fumigatus* -1.5 -1.6 -21.1 *-1.0

**C-compound and carbohydrate metabolism**

## C-compound and carbohydrate utilization

An19g00100 similar to chitinase Eh cht1 *Entamoeba invadens*  -2.5 *-1.4 *-1.9 *2.1

An16g06800 similar to endoglucanase EGLB *Aspergillus niger* *-1.5 *1.4 *-1.6 -1.9

An03g05530 similar to endo-beta-1.4-glucanase *Emericella desertoru* -2.0 *-1.0 *-1.3 *-1.2

An09g06270 similar to alcohol dehydrogenase SFA1 *1.0 *2.1 *-1.8 -1.8

An12g10140 similar to lactate dehydrogenase cytochrome b2 CYB2 *1.2 *1.0 *2.1 -7.0

An11g07330 similar to acid mono-oxygenase 6-HNAMO *Pseudomonas fluorescens* *-1.7 *1.4 *-1.5 -3.4

An02g11320 similar to UDP-glucose epimerase GAL10 *1.2 *1.3 *-1.2 -3.1

An13g01960 similar to alcohol dehydrogenase *A. fumigatus* *-1.3 *1.9 *1.5 -2.0

An09g00270 alpha-galactosidase AGLC *-1.2 -2.0 -5.9 *1.1

An09g00260 alpha-galactosidase AGLC *-2.6 -2.1 -6.0 *1.1

An02g10550 similar to endo-alpha-1.5-arabinanase ABNA *A. niger* *-1.2 -1.9 *-1.3 *-1.5

An01g01540 similar to alpha.alpha-trehalase TREA *A. nidulans* -3.0 *-1.1 -2.7 *1.2

An01g00780 endo-1.4-xylanase XYNB -3.4 *1.5 -4.3 *1.0

An05g00930 similar to mitochondrial malic enzyme MAE1 -2.1 *1.2 -4.0 *-1.0

An14g03390 similar to glutamate-ammonia lyase GLN1 -1.5 *1.1 *1.1 *-1.2

An19g00090 similar to exo-beta-1.3-glucanase CMG1 *Coniothyrium minitans* -2.6 *1.6 -2.9 *3.7

An02g00740 similar to 6-Hydroxy-D-nicotine oxidase 6-HDNO *Arthrobacter oxidans* -2.6 *1.2 *1.7 *1.4

An03g00460 similar to 6-Hydroxy-D-nicotine oxidase 6-HDNO *A. oxidans* -3.4 *-1.5 *-3.0 *1.1

An03g06270 similar to isoamyl alcohol oxidase MREA *A. oryzae* -2.7 *1.9 *-2.8 *1.0

## C-compound and carbohydrate transport

An13g02590 similar to glucose permease RGT2 *-1.0 *1.1 *1.4 -2.6

An18g04910 similar to monocarboxylate transporter MCT2 *R. norvegicus* *1.2 *-1.2 *2.4 -1.6

An16g06580 similar to hexose transporter HXT2 *-1.1 *1.5 *-1.6 -15.1

An11g04780 similar to protein involved in active glycerol uptake GUP1 -1.7 *1.2 -2.4 *1.0

**metabolism of vitamins. cofactors. and prosthetic groups**

An03g06730 similar to COBW *Pseudomonas denitrificans* *-1.7 *-1.7 *1.9 -103.8

An01g04250 similar to uroporphyrinogen decarboxylase HEM12 *-1.2 *1.1 -1.6 -1.6

## **Secondary metabolism**

An11g07310 similar to polyketide synthase PKS1 *Colletotrichum lagenarium* *1.3 *1.4 *-1.3 -4.7

An07g05520 similar to O-methyltransferase B OMTB *Aspergillus parasiticus* *-1.0 *1.0 *2.9 -2.0

An03g05210 similar to reticuline oxidase BBE1 *Eschscholzia californica*  -4.1 *-1.3 *-1.0 *1.3

## **Cellular transport**

### Import

An01g01620 similar to high-affinity zinc transport protein ZRT1 *-1.9 *1.0 *2.6 -2.3

An15g07190 similar to high-affinity zinc transport protein ZRT1 *1.7 *1.8 *1.2 -104.7

An12g10320 similar to high-affinity zinc transport protein ZRT1 *1.1 *1.4 -2.2 -3.2

An14g06280 similar to iron-regulated transporter IREG1 *Mus musculus* *-1.4 *1.2 *-1.1 -3.3

An07g06240 similar to ferrioxamine B permease SIT1 *-1.2 *1.5 *3.2 -1.5

An09g00930 similar to ABC transporter PMR1 (Ca^2+^ and Mn ^2+^ into Golgi) *-1.0 *-1.1 *1.2 -2.6

An06g01080 similar to cation diffusion facilitator MSC2 -1.8 *-1.3 *-1.5 *-1.2

An08g04670 similar to phosphate and manganese transporter PHO84 -1.6 *1.2 *-1.1 *-1.2

## Mitochondrial

An08g10600 similar to iron/sulfur ABC transporter ATM1 *-1.1 *-1.1 *-2.3 -2.2

## Vacuolar

An12g04070 similar to zinc-transporter ZRT3 *-1.7 *-1.0 *1.4 -2.8

An16g06740 similar to cadmium resistance protein YCF1 *-1.4 *-1.7 -1.7 -1.7

An03g04060 similar to cadmium resistance protein YCF1 -2.1 *-1.4 *1.2 *1.0

An15g03900 similar to heavy metal ion resistance protein ZRC1 -1.7 *1.5 *-1.9 *-1.4

## Extracellular

An06g00770 similar to HC-toxin efflux pump *Cochliobolus carbonum* *-1.1 *1.1 *5.8 -1.7

An03g00680 similar to multidrug resistance protein FNX1 *1.2 *-1.3 *1.9 -5.0

An14g00970 similar to multidrug resistance protein FNX1 *-2.3 *-1.4 *-1.1 -1.7

An04g05680 similar to metal transporter SMF1 *1.3 *1.1 *1.4 -1.7

An03g03560 similar to ferrichrome-type siderophore transporter ARN1 *-1.6 *1.7 *2.2 -2.4

#### Cell Rescue. Defense and Virulence

An05g01070 similar to 7-aminocholesterol resistance protein RTA1 *-1.2 *-1.1 *29.4 -1.6

An01g12530 similar to manganese superoxide dismutase SOD2 *-1.1 *1.1 *63.2 -1.5

An07g00570 similar to integral membrane protein PTH11 *Magnaporthe grisea* *-1.1 *-1.0 -2.7 -2.0

An18g00980 similar to integral membrane protein PTH11 *M.grisea*  *-3.0 *1.4 -4.6 -2.0

An16g05920 similar to integral membrane protein PTH11 *M. grisea*  *1.1 -3.9 -5.5 *1.0

An01g00330 alpha-l-arabinofuranosidase a precursor ABFA *-1.6 -4.6 *1.3 *1.5

#### Transcription

An07g09050 similar to hypothetical transcription regulator *Streptomyces coelicolor* *1.7 *-1.3 *22.3 -1.7

An09g06280 similar to hypothetical transcription factor *A. oryzae* *-1.3 *1.0 *-1.1 -1.5

An01g11200 similar to serine kinase SKY1 *1.2 *-1.2 *-1.0 -2.3

An08g01860 similar to zinc-transcriptional regulation ZAP1 * *-12.5 *46.3 -2.3

## **Nucleotide / nitrogen and sulfur metabolism**

An10g00800 similar to purine nucleoside permease NUP *Candida albicans*  -5.4 *-1.5 -31.9 *1.2

An12g09600 similar to allantoate permease DAL5 -1.9 *2.0 *-6.5 *1.0

An18g01220 similar to allantoate permease DAL5 -1.6 *1.6 -4.2 *-1.2

#### Other

An07g06530 similar to multicopy suppressor SUR7 *2.0 *1.7 -5.0 -1.6

An16g05910 similar to putative cytochrome P450 *A. fumigatus* *-1.2 -2.3 -5.8 *2.2

An08g07090 similar to protein SIM1 -1.7 *-1.4 *1.1 *1.4

An11g09870 similar to palmitoleyltransferase ERF2 -1.6 *1.4 -2.1 *1.0

An15g06140 similar to heterokaryon incompatibility protein HET-C *N. crassa* -2.2 *-1.3 *2.7 *1.1

# Unclassified

An02g06440 similar to molasses resistency protein RTM1 *1.2 *-1.1 *1.9 -1.8

An02g03570 similar to membrane protein YBR159w -2.1 *-1.3 *-1.9 -1.6

An01g10900 similar to hypothetical la costa protein LCS *-1.4 *3.3 *9.4 -4.6

An11g07340 similar to hypothetical O-methyl transferase ENCK *1.3 *1.3 -4.2 -4.2

An12g09640 similar to hypothetical GTP cyclohydrolase *2.6 *1.3 -3.3 -1.9

An01g09220 weakly similar to tyrosinase MELC2 *-2.1 *2.1 -5.2 -1.8

An14g01820 similar to hypothetical cell wall protein BINB *1.4 *1.2 *-1.4 -3.2

An01g03810 similar to hypothetical HIT-like protein *1.2 *1.5 *3.3 -1.6

An13g04070 similar to FLO11 gene expression regulator AT14 *-1.1 *-1.1 *3.6 -1.6

An07g07300 similar to prostaglandin f(2alpha) synthase TbPGFS *-1.3 *1.1 *6.0 -1.6

An02g08050 similar to hypothetical phosphatidyl synthase *1.0 *1.9 *3.0 -1.8

An03g00770 similar to allergic bronchopulmonary s allergen rAsp f 4 *A. fumigatus* *-2.3 *-1.1 -2.6 -2.8

An16g03330 weakly similar to endo-1.4-beta-xylanase *1.2 *-1.2 *2.1 -1.6

An01g02760 weakly similar to killer toxin KHR *-1.4 *1.3 *-1.8 -1.9

An08g02300 weakly similar to enniatin synthase *1.1 *-1.2 -2.4 -2.9

An03g06670 weakly similar to myosin-like protein MLP1 *1.1 *-1.3 *1.3 -7.3

An03g00830 weakly similar to intestinal mucin MUC2 *-1.5 *-1.4 *1.1 -2.3

An12g09870 hypothetical protein *4.2 *1.3 *2.2 -2.2

An12g10350 hypothetical protein *1.1 *1.6 *1.5 -2.2

An08g03760 hypothetical protein *-1.1 *-1.3 -4.0 -2.4

An06g00320 hypothetical protein *2.0 *-3 *1.0 -1.7

An02g08330 hypothetical protein *1.2 *-17 13.5 -1.8

An06g01000 hypothetical protein *-1.1 *-1.0 -1.83 -2.3

An11g00890 hypothetical protein *-1.1 *1.4 *-1.6 -1.5

An12g10810 hypothetical protein *1.4 *1.1 *69.5 -2.08

An03g03530 hypothetical protein *1.5 *1.2 *2.4 -1.8

An02g03100 hypothetical protein *1.2 *-1.4 *97.9 -7.3

An03g05110 hypothetical protein *-1.3 *-1.2 *1.1 -2.5

An01g01120 hypothetical protein *-1.5 *-1.8 *13.0 -2.0

An11g07020 hypothetical protein *1.3 *1.2 -2.1 -1.8

An02g08300 hypothetical protein *2.2 *-1.7 -3.2 -8.6

An03g00520 hypothetical protein *1.4 *-3.0 *12.1 -1.9

An04g04280 hypothetical protein *1.4 *1.5 *1.8 -2.2

An16g08090 hypothetical protein *-1.2 *1.2 *1.2 -1.5

An02g01320 hypothetical protein *-1.1 *1.1 *10.6 -2.0

An03g00840 hypothetical protein 1.8 *-1.2 *1.3 -2.4

An08g07150 hypothetical protein *-1.7 *-1.3 *1.2 -1.8

An11g01660 hypothetical protein *-1.8 *1.1 *1.4 -2.2

An18g01000 hypothetical protein *-2.4 *1.1 -4.6 -1.8

An14g07230 hypothetical protein *-1.2 *1.4 *-3.2 -11.0

An01g00110 hypothetical protein *-2.9 *1.4 *-1.1 -2.4

An12g02660 hypothetical protein *-1.5 *1.3 *54.9 -24.5

An01g14810 hypothetical protein *-1.3 *-1.3 *3.4 -70.2

An13g01520 hypothetical protein -1.5 *-2.9 *-1.0 -1.9

An01g14820 hypothetical protein *-2.3 *1.2 *1.1 -31.0

An07g03050 hypothetical protein *-1.1 *1.2 *2.4 -2.8

An16g05930 hypothetical protein *-1.1 -1.8 *-1.5 *-1.6

An07g05660 hypothetical protein *-1.1 -1.9 *-4.1 *-1.3

An01g02700 hypothetical protein -1.5 *-2.8 *-16.9 *-1.2

An01g04650 hypothetical protein -1.7 *-1.2 *1.0 *1.0

An01g07380 hypothetical protein -1.6 *-6.1 *-1.2 *-1.0

An02g00120 hypothetical protein -2.4 *1.2 -2.0 *-1.8

An02g13770 hypothetical protein -2.3 *-1.8 *-1.2 *1.0

An04g01690 hypothetical protein -2.1 *-1.0 -3.1 *-1.1

An05g01770 hypothetical protein -1.5 *-1.5 -5.8 *1.0

An07g05160 hypothetical protein -1.7 *-1.2 -2.1 *1.0

An07g08400 strong similarity to allergen rAsp f 4 *A. fumigatus* -1.8 *1.3 -4.2 *1.1

An08g01180 hypothetical protein -1.6 *1.3 *-1.7 *-1.2

An08g07000 hypothetical protein -1.5 *1.4 *-4.1 *1.3

An08g08600 similar to RTN2 -1.9 *-1.0 -3.3 *-1.0

An09g04870 hypothetical protein -1.5 *1.0 *1.3 *1.1

An11g00090 hypothetical protein -2.3 *1.0 *-1.9 *-1.1

An12g10590 hypothetical protein -2.6 *1.1 -12.0 *1.0

An14g00150 hypothetical protein -1.6 *1.0 *-1.8 *1.1

An14g03020 similarity to secreted protein *H. sapiens* -2.1 *1.0 *-2.8 *-1.1

An15g01740 similar to ER protein of unknown function -1.6 *1.4 -2.1 *1.0

An15g05020 hypothetical protein -3.2 *-2.2 *2.6 *1.1

The fold changes in expression are indicated for all the treatments (Tun = tunicamycin, DTT = dithiothreitol, tPA = production of t-PA) and are averaged over two experiments. Similarities are expressed in comparison with the *Saccharomyces cerevisiae* genome except when indicated. The symbol* indicates that the value does not meet one of the 3 restrictive criteria defined in the Method section.
